# Trends and ethnic disparity in endometrial cancer mortality in South Africa (1999–2018): A population-based Age-period-cohort and Join point regression analyses

**DOI:** 10.1371/journal.pone.0313487

**Published:** 2025-01-24

**Authors:** Gbenga Olorunfemi, Elena Libhaber, Oliver Chukwujekwu Ezechi, Eustasius Musenge

**Affiliations:** 1 Division of Epidemiology and Biostatistics, School of Public Health, University of Witwatersrand, Johannesburg, South Africa; 2 Faculty of Health sciences, University of Witwatersrand, Johannesburg, South Africa; 3 Division of Clinical Sciences, Nigerian Institute for Medical Research, Lagos, Nigeria; Atal Bihari Vajpayee Institute of Medical Sciences & Dr Ram Manohar Lohia Hospital, INDIA

## Abstract

**Background:**

Endometrial cancer is the sixth leading cause of cancer among females and about 97,000 global deaths of endometrial cancer. The changes in the trends of obesity, fertility rates and other risk factors in South Africa (SA) may impact the endometrial cancer trends. The aim of this study was to utilise the age period cohort and join point regression modelling to evaluate the national and ethnic trends in endometrial cancer mortality in South Africa over a 20year period (1999–2018).

**Methods:**

Data from Statistics South Africa was obtained to calculate the annual number of deaths, and annual crude and age standardised mortality rates (ASMR) of endometrial cancer from 1999–2018. The overall and ethnic trends of endometrial cancer mortality was assessed using the Join point regression model, while Age-period-cohort (APC) regression modelling was conducted to estimate the effect of age, calendar period and birth cohort.

**Results:**

During the period 1999–2018, 4,877 deaths were due to endometrial cancer which constituted about 3.6% of breast and gynecological cancer deaths (3.62%, 95% CI: 3.52%–3.72%) in South Africa. The ASMR of endometrial cancer doubled from 0.76 deaths per 100,000 women in 1999 to 1.5 deaths per 100,000 women in 2018, with an average annual rise of 3.6% per annum. (Average Annual Percentage change (AAPC): 3.6%, 95%CI:2.7–4.4, P-value < 0.001). In 2018, the overall mean age at death for endometrial cancer was was 67.40 ± 11.04 years and, the ASMR of endometrial cancer among Indian/Asians (1.69 per 100,000 women), Blacks (1.63 per 100,000 women) and Coloreds (1.39 per 100,000 women) was more than doubled the rates among Whites (0.66 deaths per 100,000 women). Indian/Asians had stable rates while other ethnic groups had increased rates. The Cohort mortality risk ratio (RR) of endometrial cancer increased with successive birth cohort from 1924 to 1963 (RR increased from 0.2 to 1.00), and subsequently declined among successive cohorts from 1963 to 1998 (1.00 to 0.09). There was strong age and cohort but not period effect among the South African women. Ethnic disparity showed that there was age effect among all the ethnic groups; Cohort effect among Blacks and Coloureds only, while Period effect occurred only among Blacks.

**Conclusions:**

The mortality rates of endometrial cancer doubled over a twenty-year period in South Africa from 1999–2018. There was strong ethnic disparity, with age and cohort effect on endometrial cancer trends. Thus, targeted efforts geared towards prevention and prompt treatment of endometrial cancer among the high-risk groups should be pursued by stake holders.

## Introduction

Endometrial cancer is the sixth leading cause of cancer among females and about 97,000 global deaths of endometrial cancer were reported in 2020 [1]. The age standardised incidence rate of endometrial cancer among high income countries (HICs) was about thrice the rate in Low-and middle-income countries (LMICs) (14.4 per thousand vs 5.5 per thousand) [2]. However, there is an increasing trend of endometrial cancer in most LMICs because of increased prevalence of major risk factors such as obesity and low fertility rates [2–4]. The overall survival rate and prognosis of endometrial cancer is generally good as the mortality to incidence ratio (MIR) is relatively low as compared to a fatal gynaecological cancer such as ovarian cancer (0.21 vs 0.64) [1]. The survival rate of endometrial cancer is more than 80% in HICs because the women present early to health facilities and prompt curative treatment can be achieved through appropriate surgery, chemotherapy and or radiotherapy by experienced gynaecological oncologist [5]. However, the survival rate among LMICs is lower because of poor awareness of symptoms, poor health seeking behaviour and sub-optimal health system [5].

Endometrial cancer may be type I (endometroid adenocarcinoma) or type II [3, 6]. Genetic predilection for endometrial cancer may be related to a family history of endometrial cancer or colorectal cancer [3, 6, 7]. Endometrial cancer is also associated with genetic disorders such as Lynch Syndrome and Cowden syndrome [3, 6, 7]. The risk factors for Endometrial cancer are related to unopposed chronic exposure of endogenous and exogenous estrogen on the endometrium [3, 6–8]. Thus, obesity, low parity, early menarche, late menopause, late age at last childbirth, polycystic ovarian syndrome and chronic use of hormone replacement therapy and Tamoxifen are risk factors of endometrial cancer [3, 5–8]. However, administration of exogenous progesterone or combined oral contraceptives and tobacco smoking is protective of endometrial cancer [2, 3, 5–8] Population based interventions can therefore target the risk factors, thereby reducing the incidence and mortality of endometrial cancer.

Endometrial cancer is the fourth most common cause of cancer deaths among South African women. South Africa is an upper middle-income country, and the country is classified as having high human development index (HDI) that increased from 0.627 in 1990 to 0.709 in 2019. South Africa is currently undergoing epidemiological and health transition especially after the commencement of muti racial democracy in 1994 [9–12]. The prevalence of obesity is rising in the South Africa, and low fertility rate is common in the country [13–16]. South Africa has one of the highest prevalence of contraceptive use of nearly 50% in sub-Saharan Africa, with majority of women accepting the injectable progesterone contraceptive [17]. Access to sexual and reproductive services and cancer care is also improving in South Africa since 1994 [11, 17]. All these shifts in risk factors of obesity over time may impact on the trends in the incidence and survival of endometrial cancer in the country. Indeed, health outcomes are generally related to ethnic disparity in access to healthcare [18]. According to Statistics South Africa (Stats SA), South Africa recognises four ethnic groups, with varying socioeconomic status and access to healthcare. The ethnic groups with their population proportion in 2021 were: Blacks (76.4%), Coloureds (mixed race) (9.1%), Whites (8.9%) and Indians/Asians (2.5%) respectively [19]. By 2018, the proportion of women aged 50 years and older among Blacks, Couloreds, Indian/Asian and Whites were 22.1%, 31.21%, 28.21% and 48.6% respectively. While the proportion of women who were 60 years and older by 2018 were 11.3%, 16.07%, 14.14% and 32.19% among Blacks, Coloureds, Indian/Asians and Whites respectively [20]. This may suggest that Female Blacks had the lowest life expectancy while Whites had the highest life expectancy in South.

Age-period-cohort (A-P-C) regression modelling is utilised to disentangle the intertwined effect of age, calendar period and birth cohort on health outcome [8, 21–24]. The age effect is usually biologic as the incidence of some diseases become pronounced with increasing age and ageing [8, 23, 24]. On the other hand, period effect is due to population based interventions and programs that affect all age groups [8, 23, 24]. The deployment of novel diagnostic tools and treatment for endometrial cancer are also calendar period effect that can influence endometrial cancer trends over time. Improvement or disruptions in cancer registrations, coupled with changes in disease classification can impact on observed cancer trends and these are captured based on the period effect [8, 23, 24].

The birth cohort effects are cohort specific risk of a disease that is related to the social, reproductive and environmental exposure of a group of people that were born around the same time [8, 23, 24]. To our knowledge, no study has utilised age period cohort analysis to evaluate the national and ethnic burden of endometrial cancer mortality in Sub-Saharan Africa. We therefore utilised the age period cohort and join point regression modelling to evaluate the national and ethnic trends in endometrial cancer mortality in South Africa over a 20-year period (1999–2018). The evidence from this study can highlight important patterns and trends of endometrial cancer to guide policy makers.

## Methodology

### Study design and data source

This study was a cross-sectional and temporal secondary data analysis. Data for endometrial cancer deaths was obtained from Statistics South Africa (Stats SA). The Stats SA collects and publishes anonymized mortality data in South Africa. The Stats SA data also contains the age, year of death and ethnicity of the decedents [25, 26]. Causes of death were coded using the International Classification of Diseases, Tenth Revision (ICD-10) [27]. The code for the underlying cause of death for endometrial cancers was ICD10, C54 [26]. Mid-year population estimates of females (≥15 years) stratified by ethnicity and 5-year age group were obtained from Stats SA from 2002 to 2018. A constant inter-censal population growth rate was assumed between 1996 and 2001 when national censuses were conducted in South Africa.

#### Data quality

The vital registration data of South Africa has been assessed to be one of the three high quality data in SSA [28]. The coverage, completeness, temporal consistency, timeliness and sub-national availability of the Stats SA data has been adjudged to be of high standards [26, 28]. The Stats SA vital statistics data is the only nationally representative cancer mortality records in South Africa.

*Ethical considerations*. Before commencement of the study, ethical approval was obtained from the Human Research Ethics Committee (Medical) of the University of the Witwatersrand (Clearance certificate number: M190544). Confidentiality was ensured as anonymized data was utilized.

#### Statistical analysis

Stata version 17 (Statacorp, USA) statistical software was utilised for statistical analysis. The frequency of categorical variables and mean (±standard deviation) of continuous variables were conducted. The annual proportion of endometrial cancer in relation to all female breast and gynaecological cancer mortality was calculated. The annual crude mortality rate (CMR) of endometrial cancer was calculated by dividing the annual deaths among women aged ≥15 years by the mid-year female population (≥15years). Age specific mortality rate was also calculated by dividing the cumulative age stratified mortality of each 5-year age group (15–19, 20–24, 25–29……..75+) by cumulative age stratified mid-year population of each age category. The Annual age standardised mortality rates (ASMR) were calculated using the direct method of standardisation, based on the 1964 Segi world standard population as weighted population. All rates were stratified by ethnicity (Blacks, Coloureds, Whites, Indian/Asian) and expressed per 100,000 women. Microsoft Office Excel was used for calculating and producing graphs of annual CMRs and ASMRs.

#### Join point regression modelling of endometrial cancer

The estimated annual percent change (EAPC) and average annual percent change (AAPC) of the ASMR of endometrial cancer from 1999–2018 was estimated using the Joinpoint Regression software, version 4.9.1.0 (Statistical Methodology and Applications Branch, Surveillance Research Program, National Cancer Institute, Bethesda, MD). The Join point regression software fits a Poisson regression in which the natural logarithm of ASMR is the outcome and the year of death was the predictor variable. Four maximum Join points and 4499 Monte Carlo permutation tests were conducted for the trends. The segmental EAPC was calculated as

$$(exp\left(\beta \right)-1)\times 100$$

[29].

The AAPC of the overall trends were calculated as the average of all the segmental EAPCs. Positive, or negative AAPC with P-value <0.05 was taken as a statistically significant increase or decrease. AAPCs with P-value >0.05 was presented as a non-significant increased or decreased trend. AAPCs from -0.5 to + 0.5 with p-value >0.05 were reported as stable trends [29]. Similar trends analysis was conducted for each of the 5-year age group and each ethnic group.

#### Age period cohort modelling of endometrial cancer mortality

To examine the effect of age, period and birth cohort on endometrial cancer mortality, the age period cohort (APC) regression modelling was performed. A lexis matrix was formed with 5-year age category (15–19 years, 20–24 years, 25–29 years, 30–34 years, 35–39 years……75+) as columns and the corresponding 5-year calendar period (1999–2003, 2004–2008, 2009–2013, 2014–2018) as rows. The diagonal will be the corresponding birth cohort. The lexis matrix was imputed into the Age-period-cohort Web Tool (Biostatistics Branch, National Cancer Institute, Bethesda, MD, USA). (Age Period Cohort Analysis Tool (cancer.gov)) to produce estimable parameters that is based on weighted least squares estimator [23]. The A-P-C model assumes a Poisson distribution of the mortality rates (dependent variable) with age, period and birth cohort as the independent variables.

Thus,

$$\text{Age}\;\text{at}\;\text{death}=\text{period}\left(\text{year}\;\text{of}\;\text{death}\right)–\text{Birth}\;\text{cohort.}$$

Since age, calendar period and birth cohort are perfectly linear and dependent, identifiability problem will occur when the three variables are covariates in a model. The identifiability problem was circumvented by the APC web tool and estimable parameters were calculated [23, 24]. The estimable parameter include: (1) Net drift (equivalent to the AAPC) of the ovarian cancer mortality from 1999–2018. (2) local drift (equivalent to the annual percent change per age group) (3) Cohort rate ratio (RR) (4) Period RR (5) longitudinal age specific rates (6) cross-sectional age specific rates (age trend − period trend). The reference for the period and cohort estimates were 2004–2008 and 1959–1963 respectively which were the middle values. Wald’s test of statistical significance, of all the estimates were also reported. Two-tailed test of significance was assumed and P-value< 0.05 was taken as statistically significant level.

## Results

During the period 1999–2018, 4,877 deaths were due to endometrial cancer (out of 134,788 breast and gynecological cancer deaths), which constituted about 3.6% of breast and gynecological cancer deaths (3.62%, 95% CI: 3.52%–3.72%) in South Africa. About two third of endometrial cancer deaths occurred among the Blacks (N = 2,875, 69.44%)) followed by the deaths among Whites (n = 579, 13.99%) S1 Table in S2 File.

### Trends in endometrial cancer mortality 1999–2018

**Fig 1:**
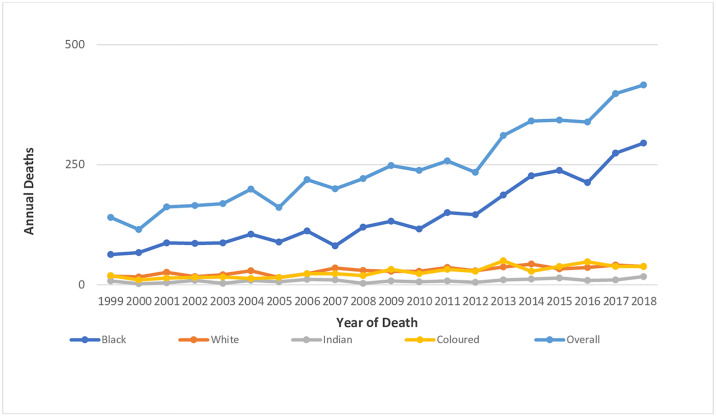
Trends in national and ethnic annual deaths of endometrial cancer.

Endometrial cancer deaths increased from 140 deaths in 1999 to 416 deaths in 2018 at about 6.1% per annum (AAPC: 6.1%, 95%CI: 5.3%–6.8%, P-value < 0.001) (Fig 1 and Table 1).

**Table 1:** Trends in the mortality rates and mean age at death of endometrial cancer in South Africa (1999–2018).

Year	Mortality from breast and gynecological cancers Frequency (%)	Mortality from endometrial cancer Frequency (%)	(% of gynecological and Breast cancer)	Age mean ± SD	CMR	ASMR
1999	4,617 (3.43)	140 (2.87)	3.03	64.67 **±** 12.57	0.91	0.76
2000	4,756 (5.53)	115 (2.36)	2.42	66.46 **±** 12.53	0.73	0.62
2001	5,124 (3.80)	162 (3.32)	3.16	66.43 **±** 12.20	1.00	0.85
2002	5,203 (3.86)	165(3.38)	3.17	66.39 **±** 12.40	1.01	0.85
2003	5,361 (3.98)	169(3.47)	3.15	66.26 **±** 13.54	0.99	0.81
2004	5,877 (4.36)	199 (4.08)	3.39	67.48 **±** 13.00	1.20	1.17
2005	6,036 (4.48)	161 (3.30)	2.67	65.75 **±** 11.35	0.99	0.85
2006	6,081 (4.51)	219 (4.49)	3.60	65.99 **±** 10.90	1.33	1.14
2007	6,199 (4.60)	200 (4.10)	3.23	68.05 **±** 12.04	1.20	0.98
2008	6,252 (4.64)	221 (4.53)	3.53	65.77 **±** 11.48	1.26	1.1
2009	6,698 (4.97)	248 (5.09)	3.70	67.13 **±** 11.90	1.40	1.2
2010	6,776 (5.03)	238 (4.88)	3.51	66.97 **±** 10.47	1.32	1.14
2011	7,096 (5.26)	258 (5.29)	3.64	67.24 **±** 11.54	1.42	1.18
2012	7,185 (5.33)	234 (4.80)	3.26	67.20 **±** 11.39	1.27	1.04
2013	7,551 (5.60)	311 (6.38)	4.12	67.21 **±** 11.11	1.60	1.28
2014	8,190 (6.08)	341 (6.99)	4.16	68.71 **±** 10.67	1.74	1.33
2015	8,427 (6.25)	343 (7.03)	4.07	67.47 **±** 11.08	1.73	1.37
2016	8,900 (6.60)	339 (6.95)	3.81	68.75 **±** 10.58	1.68	1.33
2017	9,087 (6.74)	398 (8.16)	4.38	68.04 **±** 10.54	1.94	1.51
2018	9,362 (6.95)	416 (8.53)	4.44	67.40 **±** 11.04	1.98	1.5
Total	134,778 (100.00)	4877 (100.00)	3.62	66.97 ±11.36	1.34	1.10

CMR: Crude mortality rate, ASMR: Age standardized mortality rate; SD: Standard deviation.

**Fig 2:**
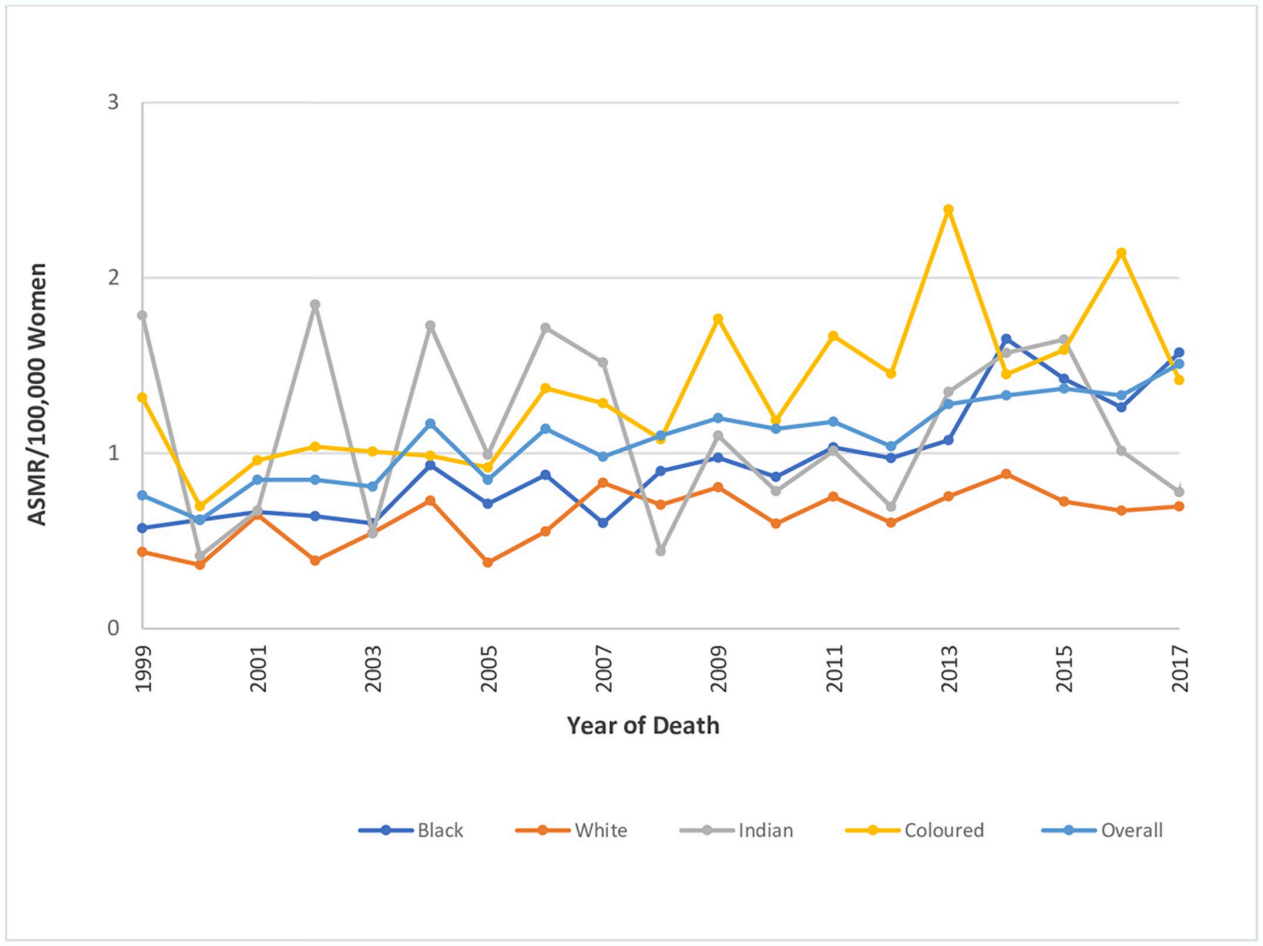
Trends in national and ethnic age standardized mortality rates of endometrial cancer.

**Fig 3:**
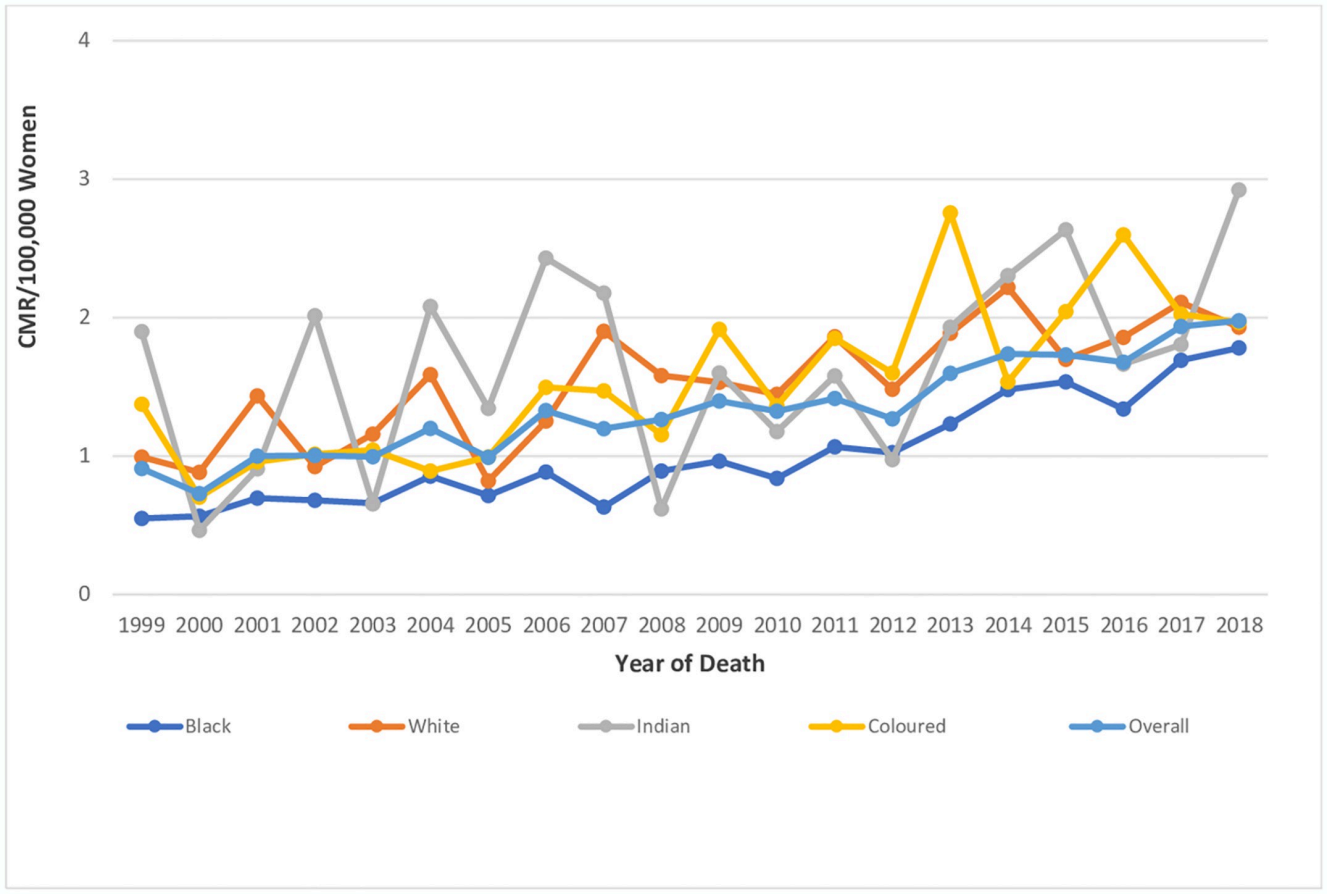
Trends in national and ethnic crude mortality rates of endometrial cancer.

**Fig 4:**
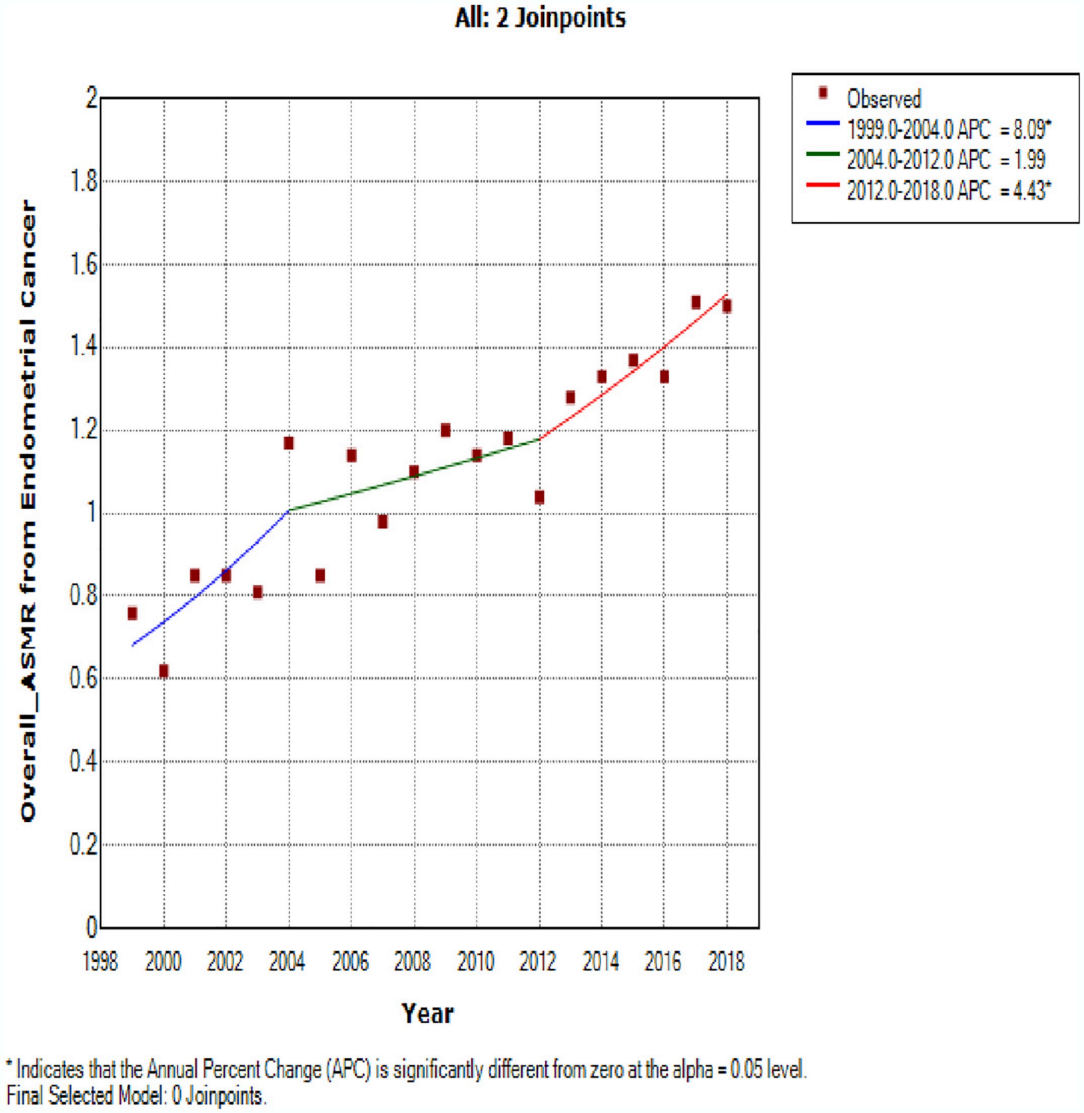
Joinpoint regression trends of the overall annual age standardized mortality rate of endometrial cancer in South Africa (1999–2018).

The ASMR of endometrial cancer doubled from 0.76 deaths per 100,000 women in 1999 to 1.5 deaths per 100,000 women in 2018, with an average annual rise of 3.6% per annum. (AAPC: 3.6%, 95%CI:2.7–4.4, P-value < 0.001) (Figs 2–4 and Table 1).

Join point regression analysis of endometrial cancer ASMR showed three trends: The first was a statistically significant increased trends of about 8.1% per annum from 1999 to 2004 (APC: 8.1%, P-value <0.001). Subsequently there was a non-statistically significant lower annual rate of 2.0% from 2004 to 2012 (APC: 2.0%, P-value = 0.2). The third trend showed a statistically significant increase of 4.4% per annum from 2012–2018 (Fig 4 and Table 2).

**Table 2:** Join point regression estimates of the trends in age standardised mortality rates of endometrial cancer in South Africa (1999–2018).

Cancer Type	Trends	Year Period	APC	95% CI	P-value	Comment
**Endometrium**						
Overall ASMR						
	1	1999–2004	8.1*	1.2–15.4	<0.001	Statistically significant increase
	2	2004–2012	2.0	-1.4–5.5	0.2	Non-statistically significant decrease
	3	2012–2018	4.4*	0.7–8.4	<0.001	Statistically significant increase
	Full Range	1999–2018	3.6*	2.7–4.4	<0.001	Statistically significant increase
Blacks	1	1999–2010	4.4*	0.8–8.1	<0.001	Statistically significant increase
	2	2010–2018	7.5*	3.0–12.2	<0.001	Statistically significant increase
	Full Range	1999–2018	5.8*	4.5–7.0	<0.001	Statistically significant increase
Indian/Asian						
	1	1999–2011	-2.6	-10.4–5.9	0.5	Non-Statistically significant decrease
	2	2011–2018	4.9	-12.3–25.4	0.6	Non-Statistically significant increase
	Full Range	1999–2018	-0.2	-3.5–3.2	0.9	Stable
Coloured						
	1	1999–2018	3.5*	1.5–5.5	<0.001	Statistically significant increase
	Full Range	1999–2018	3.5*	1.5–5.5	<0.001	Statistically significant increase
White						
	1	1999–2007	6.5	-2.1–15.8	0.1	Non-Statistically significant increase
	2	2007–2018	-0.9	-5.1–3.5	0.7	Non-Statistically significant decrease
	Full Range	1999–2018	2.2	-1.8–6.3	0.3	Non-Statistically significant increase

*Statistically significant level at P-value <0.05;

APC: Annual percent change; CI: Confidence interval.

The CMR of endometrial cancer also increased from 0.91 deaths per 100,000 women to 1.98 deaths per 100,000 women and the CMR was higher than the ASMR throughout the study period (Figs 2 and 3 and Table 1).

### Ethnic trends of endometrial cancer mortality

**Fig 5:**
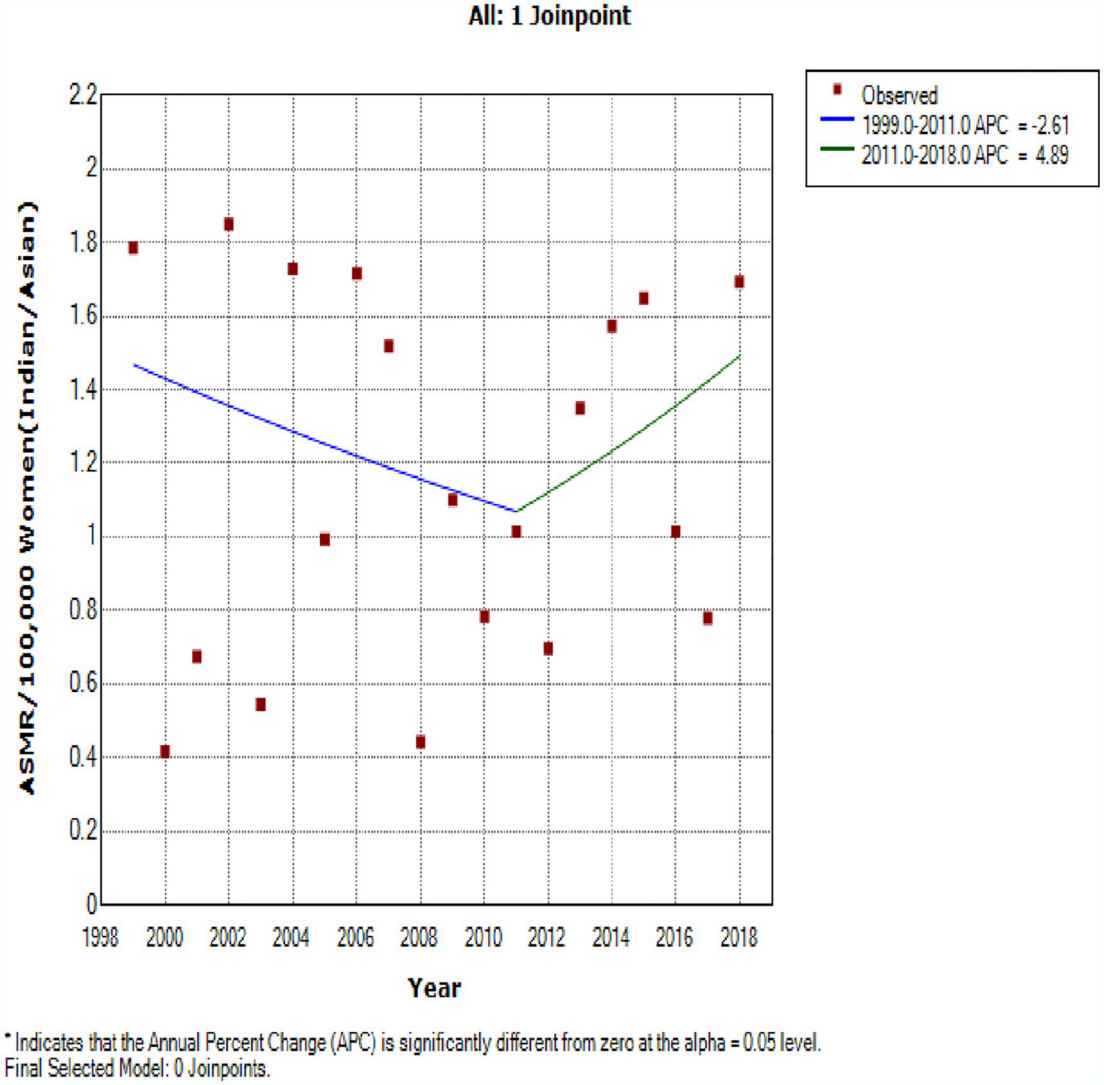
Joinpoint regression trends of the annual age standardized mortality rate of endometrial cancer in South Africa (1999–2018) among Indian/Asian ethnic group.

**Fig 6:**
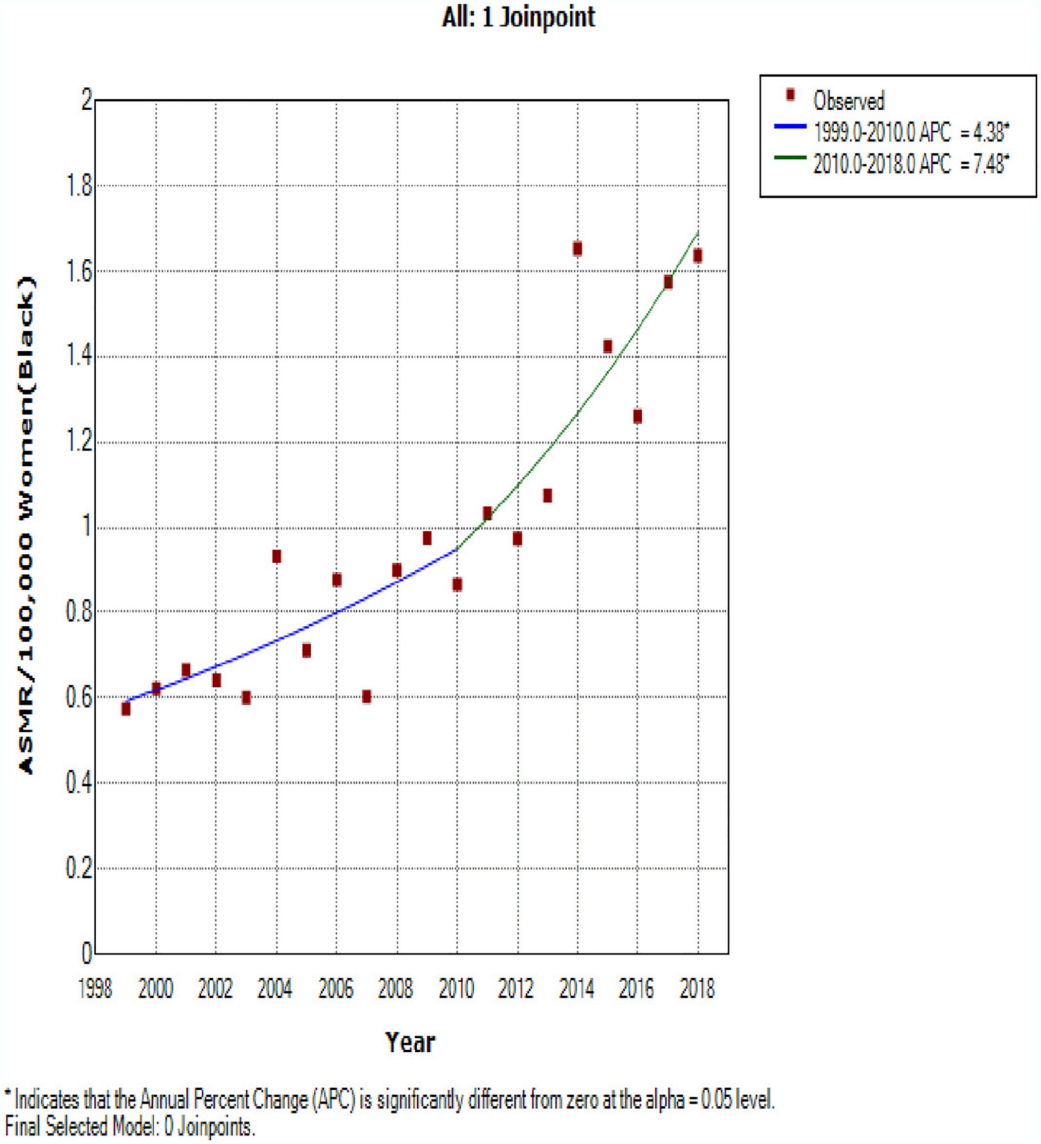
Joinpoint regression trends of the annual age standardized mortality rate of endometrial cancer in South Africa (1999–2018) among Black ethnic group.

**Fig 7:**
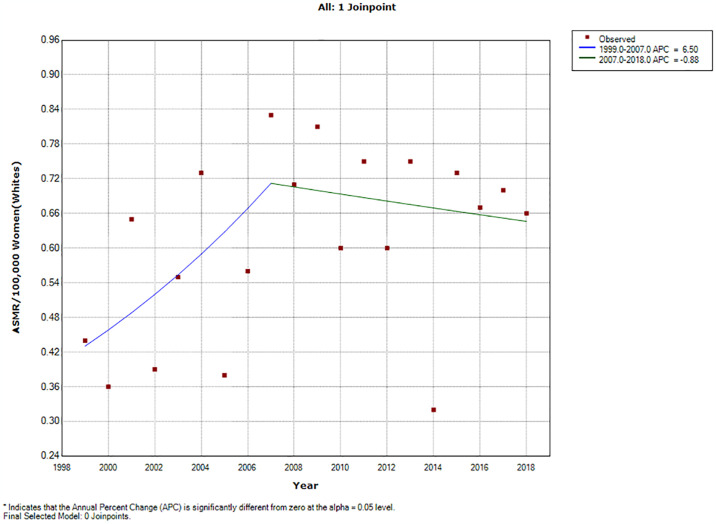
Joinpoint regression trends of the annual age standardized mortality rate of endometrial cancer in South Africa (1999–2018) among White ethnic group.

**Fig 8:**
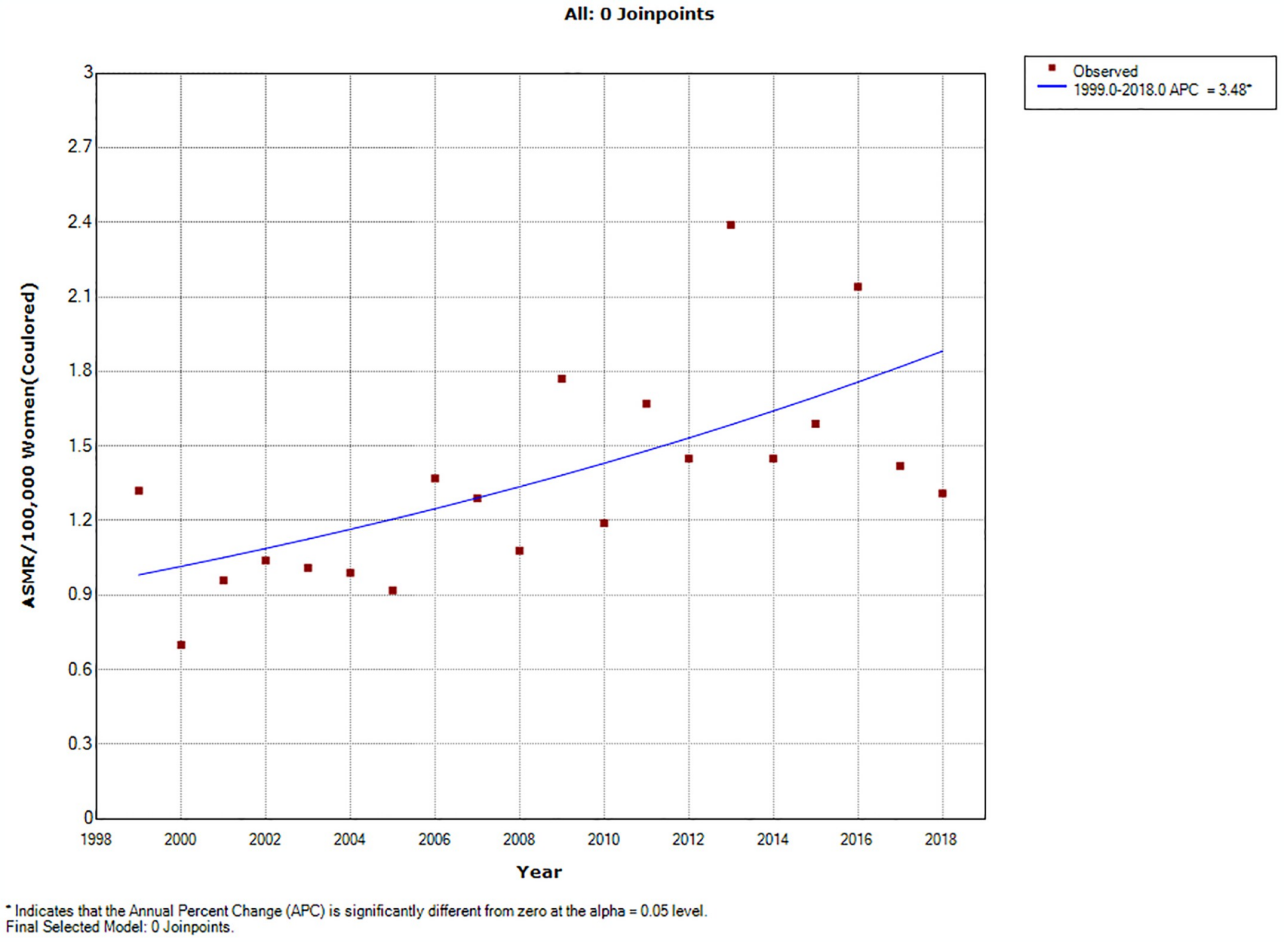
Joinpoint regression trends of the annual age standardized mortality rate of endometrial cancer in South Africa (1999–2018) among Colored ethnic group.

In 2018, the ASMR of endometrial cancer among Indian/Asians (1.69 per 100,000 women), Blacks (1.64 per 100,000 women) and Coloreds (1.31 per 100,000 women) were more than doubled the rates among Whites (0.66 deaths per 100,000 women) (S2 Table in S2 File). Indian/Asians had stable ASMR (AAPC: -0.2, P-value = 0.9) from 1999–2018 while Blacks (AAPC:5.8%, P-value < 0.001), and Coloreds (AAPC: 3.5%, P-value <0.001) had increased rates with Whites (AAPC: 2.2, P-value = 0.3) having non-significant increase (Table 2 and Figs 2 and 5–8). Notably, the join point regression of the latest segmental trends showed that White women had a non-statistically significant slight decline in annual trends from 2007 to 2018 (APC: -0.9%, P-value = 0.7), while Indian/Asians had non-significant annual increase from 2011 to 2018 (APC: 4.0%, P-value = 0.6) and Blacks had a more rapid increase from 2010–2018 (APC: 7.5%, P-value < 0.001) (Fig 2 and Table 2). The Blacks had the lowest CMR (1.78 per 100,000 women) while Indian/Asian (2.92 per 100,000 women) had the highest CMR in 2018 (Fig 3 and S2 Table in S2 File).

### Trends in mean age and age specific rates and by ethnicity of endometrial cancer

In 2018, the mean age at death from endometrial cancer in South Africa was 67.40 ± 11.04 years, and it increased from 64 years to 67 years between 1999 and 2018 (Table 1). In 2018, Whites (70.97± 9.70), but mean age at death among Coloreds (68.18 ± 9.48 years), Blacks (67.18 ±11.30 years) and Indian/Asian (60.05 ± 10.93 years) were in the sixth and seventh decades respectively (S2 Table in S2 File). The mean age at death from endometrial cancer apparently increased among the Blacks (60 year to 67 years) while the mean age fluctuates among the other ethnic groups.

### Join point trends in the overall age specific mortality rates of endometrial cancer, 1999–2018

**Fig 9:**
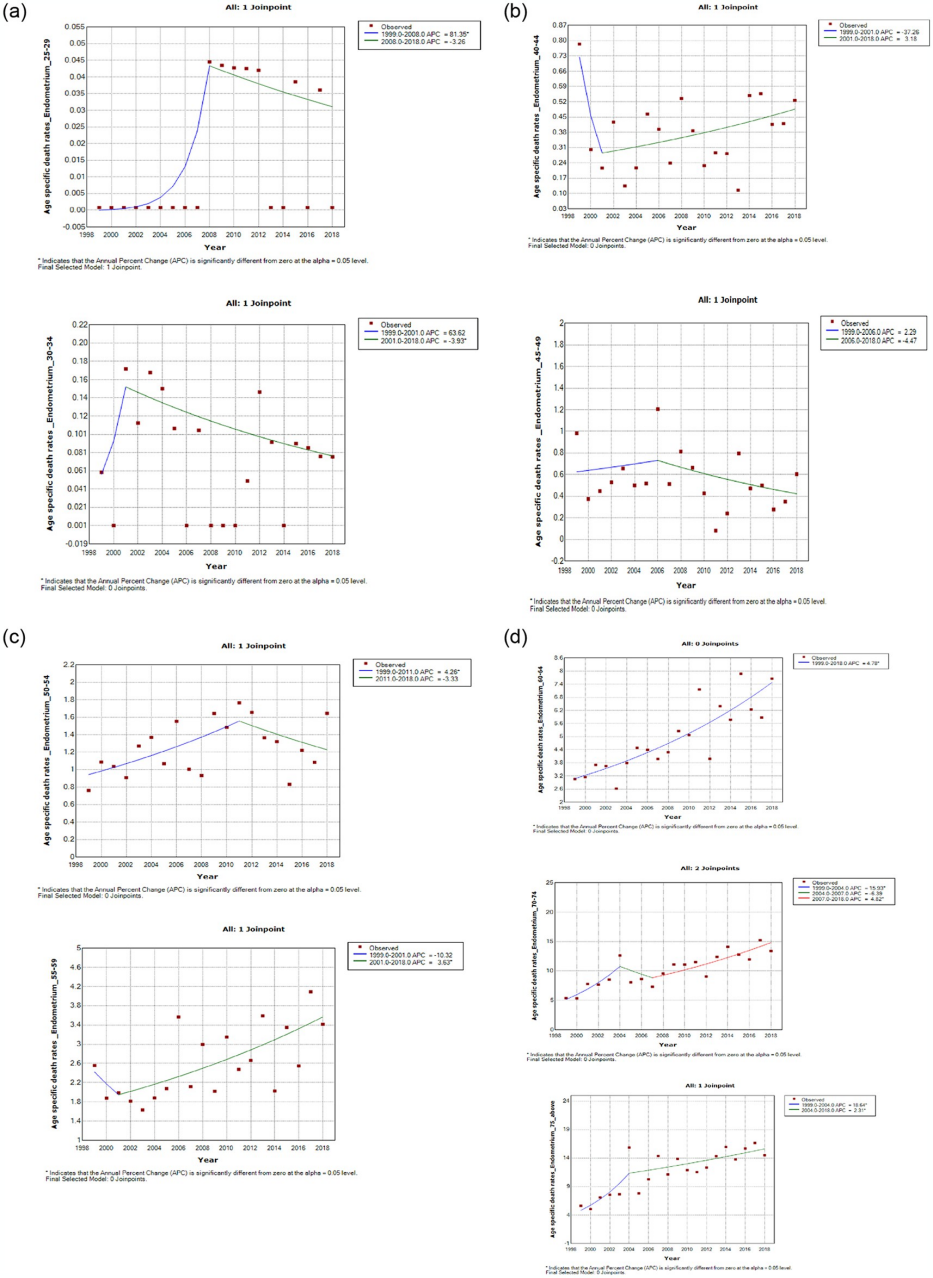
Join point trends of age specific death rates of endometrial cancer in South Africa 1999–2018.

From 1999–2018, women aged 55 years and older had statistically significant increase in annual mortality rates of endometrial cancer (AAPC range: 3.0%–4.8%, P-value < 0.001). However, there was a non-statistically significant increase in annual mortality rates of endometrial cancer among women aged 25–29 years and 45–54 years (AAPC: 1.7–5.2, Pvalue > 0.05) while there was non-significant decline in annual mortality rates among women aged 20–24 years and 30–39 years (AAPC: -8.1% to -2.9%, P-value > 0.05). Women aged 4044 years had stable trend (AAPC: 0.1, P-value = 1.0) (S3 Table in S2 File and Fig 9).

### Age specific death rate of endometrial cancer by ethnicity

**Fig 10:**
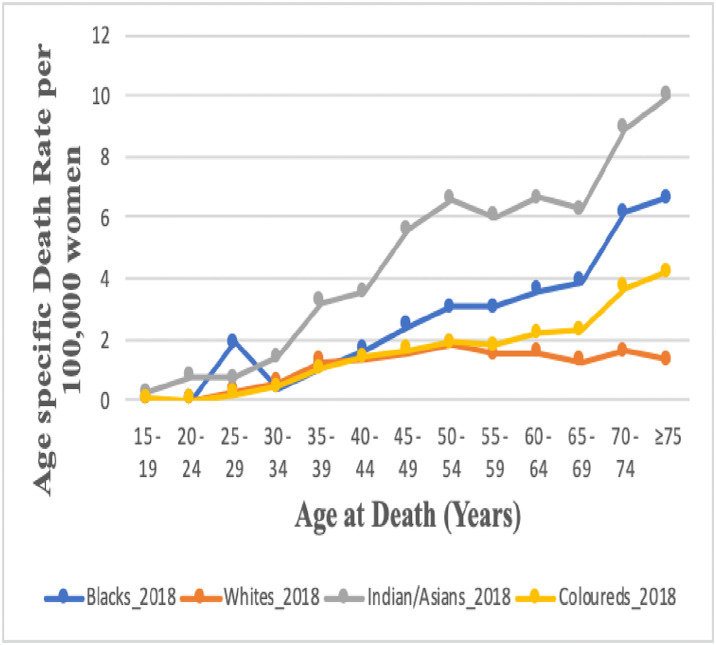
Trends in national and ethnic age specific mortality rates of endometrial cancer in South Africa (1999–2018).

In 2018, the White ethnic group had the lowest mortality rate of Endometrial cancer in all age groups in all age groups with a peak at 50–54 years, while the Indian/Asian had the highest mortality rates in all age groups followed by the black ethnic groups. The mortality rates of endometrial cancer increaed with age among the other three ethnic groups apart from Whites (Fig 10 and S4 Table in S2 File).

### Age period cohort analysis of overall and ethnic trends in endometrial cancer mortality

#### Local and net drift

**Fig 11:**
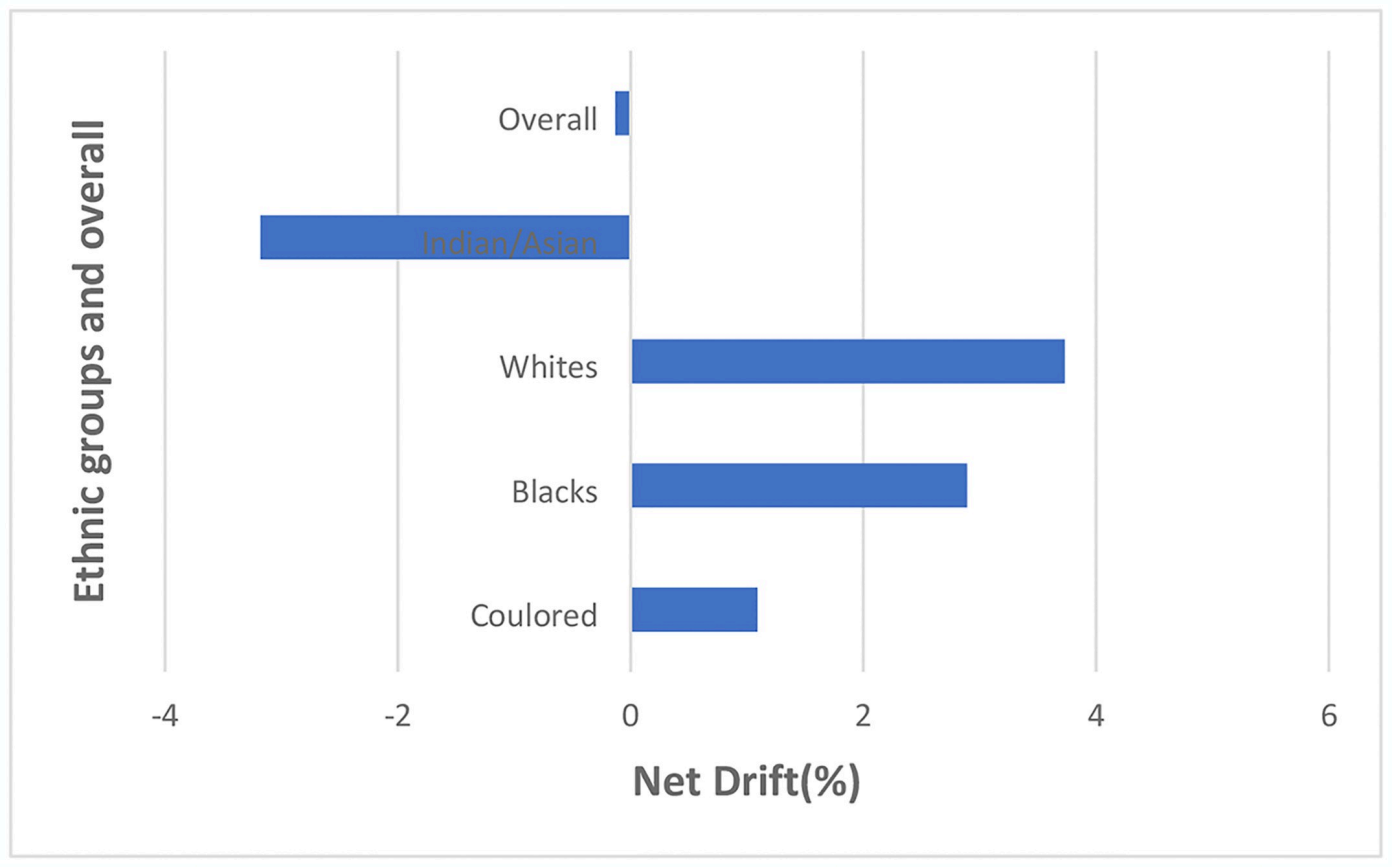
Overall and ethnic net drift of endometrial cancer mortality in South Africa (1999–2018).

The overall net drift of endometrial cancer trends from 1999–2018 was -0.13% per annum (95%CI: -2.85% to 2.67%) (S5 Table in S2 File and Fig 11 and S1 Fig in S1 File), There was positive net drift among Whites (3.73%, 95%CI: -2.01% to 9.80%), Blacks (2.89% 95%CI: -0.17% to 6.04%) and Coloreds (1.09%, 95%CI: -4.37% to 6.87%) while Indian/Asians (-3.18%, 95%CI: -9.96% to 4.12%) had negative drift (S6 Table in S2 File and Fig 11 and S2–S5 Figs in S1 File). However, the overall and ethnic net drift were not statistically significant (P-value>0.05) (S7 Table in S2 File).

**Fig 12:**
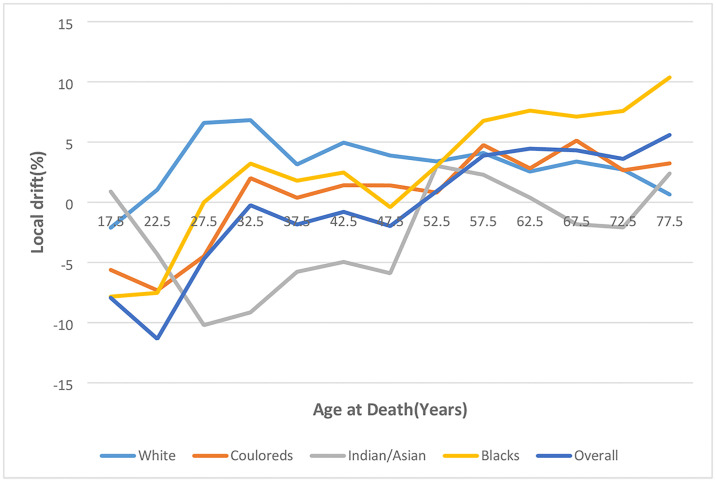
Overall and ethnic local drift of endometrial cancer mortality in South Africa (1999–2018).

The local drift of endometrial cancer mortality among women of the reproductive age (15–49 years) was less than 0, with very modest value (-0.25% to -1.98%) among women aged 30–49. Women aged 50 years and older had increasing local drifts. However, all local drifts were not statistically significant Fig 12 and S5 Table in S2 File and S1 Fig in S1 File.

The local drift among Whites was >0 among those older than 20 years and was the highest ethnic drift among women younger than 55 years. Whites aged 30–34 years had the highest local drift (6.82%, 95%CI: -11.27to28.59) per annum and there was a subsequent gradual decrease to 0.64% (95%CI: -1.58to2.91) among women aged 75 years and older Fig 12 and S6 Table in S2 File and S2 Fig in S1 File.

Young Blacks (<30 years) and those aged 45–49 years had local drift < 0 for endometrial cancer mortality. The local drift among Blacks was the second highest from 20–54 years but became the highest from 55 years. The local drift increased with age from -7.84%, (95%CI: -42.50% to 47.71%) per annum among women aged 15–19 years to 10.37%, (95%CI: 8.62% to 12.16%) per annum among women aged among women aged 75 years and older Fig 12 and S6 Table in S2 File and S3 Fig in S1 File.

Indians/Asians aged 20–49 years, 60–74 years and Coloreds aged 15–29 years had local drift of endometrial cancer < 0. The local drift among Indians/Asians generally increased from -4.32% (95%CI: -35.47% to 41.88%) per annum among women aged 20–24 years to 3.02% (95%CI: -5.04% to 11.77%) per annum among women aged 50–54 years. There was subsequent decline with age to -2.09%, (95%CI: -6.51% to2.53%) per annum among women aged 70–74 years. Women aged 75 years and older had an increased drift of 2.39%, (95%CI: -4.96 to 10.31) per annum. The local drift among Indians/Asians was generally the lowest from 25–74 years Fig 12 and S6 Table in S2 File and S4 Fig in S1 File.

The local drift of endometrial cancer mortality among Coloreds generally increased (with some fluctuations) from -7.32% (95%CI: -34.72% to 31.57%) per annum among 20–24-year-olds to 3.23% (95%CI: 0.08% to 6.47%) per annum among women aged 75 years and older. The local drift among Coloreds was generally the second lowest with similar pattern to the drifts among blacks from 20–54 years but subsequently became the second highest drift from 5 years Fig 12 and S6 Table in S2 File and S5 Fig in S1 File. The local drifts among Blacks aged 50 years and older; Whites aged 55–59 years, and 65–69 years; Coloureds aged 55–59 years, 65–69 years and 75 years and older were statistically significant. No local drift was statistically significant among Indians/Asians S6 Table in S2 File.

#### Age effect

**Fig 13:**
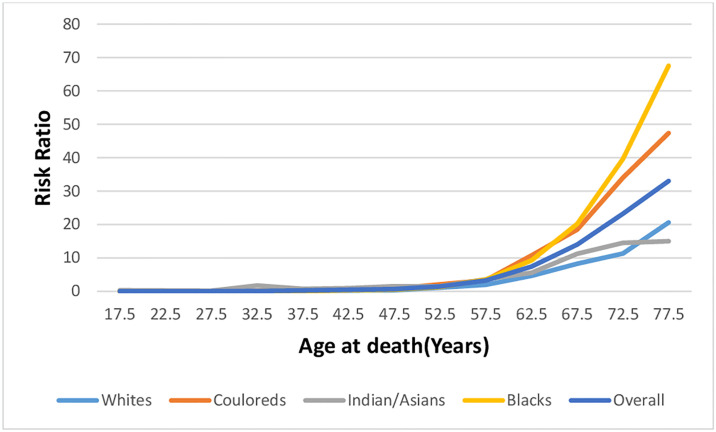
Age effects of the overall and ethnic trends of endometrial cancer mortality in South Africa (1999–2018).

Based on the longitudinal age curve, the national RR of endometrial cancer mortality increased with age, with an exponential increase from 50 years, while exponential increase occurred from 50 years for Blacks and 55 years among non-Blacks (Fig 13 and S1–S5 Figs in S1 File and S6 Table in S2 File). The Blacks followed by Whites had the lowest RR of endometrial cancer mortality with age while Couloureds and Indian/Asians had the highest RR with increasing age Fig 13 and S1–S5 Figs in S1 File and S5 and S6 Tables in S2 File.

#### Period effect

**Fig 14:**
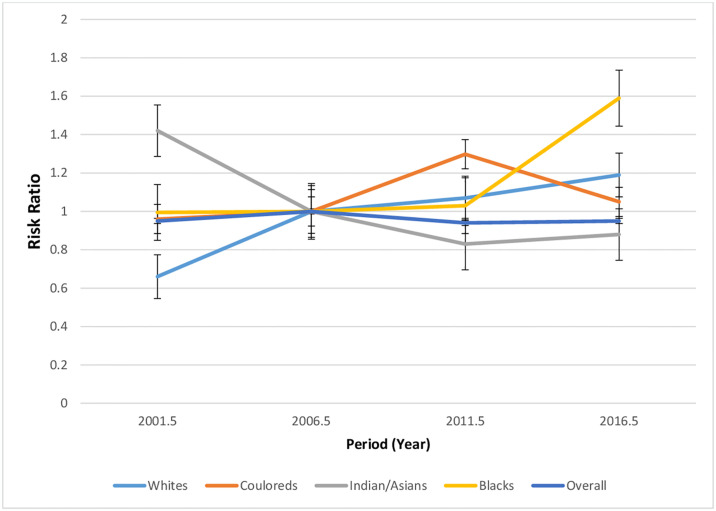
Period effects of the overall and ethnic trends of endometrial cancer mortality in South Africa (1999–2018).

The South African period RR for endometrial cancer mortality appears generally stable from 1999–2018, although there was an initial slight increase from 1999–2003 to 2004–2008 period (0.95 to 1.00) with a subsequent decline from 2004–2008 to 2009–2018 (1.00 to 0.95) Fig 14 and S1 Fig in S1 File and S5 Table in S2 File.

Blacks had stable period RR from 1999–2013 and subsequently had increased RR from 2014 to 2018. Whites had increased period RR, while Indians/Asians had declining period RR. Couloureds had increased period RR from 1999 to 2013 and a subsequent decline (Fig 14 and S2–S5 Figs in S1 File and S6 Table in S2 File. The Wald’s test for period effect of endometrial cancer mortality for the country and non-Blacks was not statistically significant. Only Blacks had a statistically significant period RR (S7 Table in S2 File).

**Fig 15:**
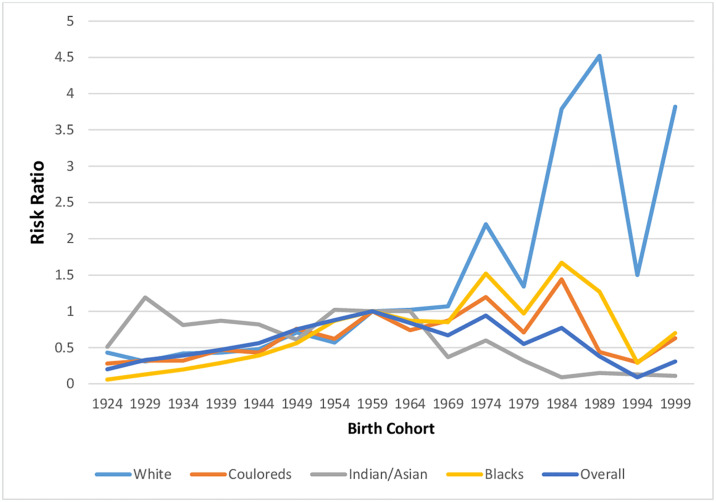
Cohort effects of the overall and ethnic trends of endometrial cancer mortality in South Africa (1999–2018).

#### Cohort effect

There was increased RR among successive cohorts from 1924 to 1963 (RR increased from 0.2 to 1.00), subsequently there was a reduction in RR among successive cohorts from 1963 to 1998 (1.00 to 0.09). There was a surge in risk among the youngest cohort (Fig 15 and S5 Table in S2 File and S1 Fig in S1 File).

The Blacks (RR:0.06, 95%CI: 0.05–0.09), Coloureds (RR:0.28, 95%CI: 0.13–0.59) and Whites (RR:0.43, 95%CI: 0.21–0.85) had low RRs among the oldest birth cohorts of 19241928, with subsequent increased mortality risk among successive birth cohorts. Recent White Cohorts had exaggerated risks from 1984–1988 birth cohort. However, there was a decline in RR among successive cohorts of Blacks and Coloureds from 1984–1998. The youngest cohorts (1999–2003) of Blacks, Whites and Coloureds had a surge in risk (Fig 15 and S2–S5 Figs in S1 File and S6 Table in S2 File).

Indian/Asians had the highest RR (0.51 95%CI: 0.11–2.40) among women born in 1924–1928 and the RR generally increased among successive cohorts till 1964–1968, after which successive cohort experienced declining RR till the most recent birth cohort (1999–2003), becoming the least ethnic specific RR among the cohorts (Fig 15 and S2–S5 Figs in S1 File and S6 Table in S2 File). The Wald’s test showed that the cohort effect was statistically significant for overall and only among Blacks and Coloreds (S7 Table in S2 File).

## Discussions

We utilized for the first time in sub-Saharan Africa both Join point regression and age, period cohort modelling to evaluate the trends in endometrial cancer mortality from 1999–2018, to provide evidence to aid targeted interventions.

### Endometrial cancer mortality trends

The mortality rate of endometrial cancer in South Africa (1.5 per 100 000 women) was lower than the average rate of 2–3 deaths per 100,000 women among Western African countries, and HICs [1]. However, the MIR of endometrial cancer in South Africa (1.5 vs 5.2 per 100,000 women, MIR:0.29) was lower than the MIR among West African countries (1.2 vs 3.3 per 100,000 women, MIR: 0.36), but doubled the MIR in North America (3 vs 21.1 per 100,000 women, MIR:0.14) [30, 31]. Thus, high endometrial cancer incidence does not necessarily translate to high mortality rate if endometrial cancer patients present at early stage and had access to optimum oncological care.

Similar to the mortality trends in some LMICs, we found that the mortality rates of endometrial cancer doubled from 0.76 deaths per 100,000 women in 1999 to 1.5 deaths per 100,000 women in 2018 at an annual rise of 3.6% per annum. In contrast, the global mortality rate of endometrial cancer declined from 1990 to 2019, despite a rise in incidence over the same period. The mortality decline occurred largely in HICs and some LMICs, because of increased awareness, early presentation, and improvement in access to surgical and chemoradiation therapy. The increased mortality rate of endometrial cancer in South Africa suggested that there was increased incidence of endometrial cancer, (fueled by increased prevalence of risk factors such as obesity and low fertility rate), with no commensurate access to optimum oncological care. Although, there was expansion of access to reproductive health services in South Africa after the commencement of multi racial democracy in South Africa in 1994, these interventions have not been able to curtail the apparent rising increase in endometrial cancer risks and incidence in the country [9, 11, 32, 33]. Thus, the period RR was relatively unchanged and not statistically significant from 1999–2018. However, tobacco smoking control policies and improvement in the diagnosis and quality of death registration of endometrial cancer after commencement of multi-racial democracy in 1994 can partly contribute to the increase in the endometrial cancer incidence and mortality in the country [34].

### Age effect of endometrial cancer mortality trends

We reported strong age effect on endometrial cancer mortality in South Africa. Endometrial cancer mortality risk increased with age and there was an exponential increase from 50 years, possibly because majority of endometrial cancer cases occurred during the post-menopausal period. In line with previous studies, that reported the average age of endometrial cancer mortality to be in the sixth and seventh decades, the average age of endometrial cancer death in South Africa increased from 64 years in 1999 to 67 years in 2018. The observed increased age at endometrial cancer death may suggest some improvement in survival rate at the population level, possibly because of increased awareness and improved access to reproductive healthcare services in the country. We observed that women younger than 50 years generally had negative local drifts possibly because they had more access to the expanded reproductive health services during childbirth, post-partum, family planning visits and other sundry reproductive health and gynecological visits. Young women may also have better health seeking behavior, be educated and aware of endometrial cancer symptoms. They may also benefit from regular health screening programs in the workplace. Furthermore, the protective effect of hormonal contraceptives will be more apparent among young women of the reproductive age group. The increased mortality rate of endometrial cancer among women older than 70 years may be linked to increased life expectancy and associated co-morbidities at older age as the life expectancy is generally increasing in South Africa [35–39]. Furthermore, endometrial cancer at younger age tends to have better prognosis.

### Cohort effect of endometrial cancer mortality

In line with reports from USA and Eastern Asia, Japan, China, we reported that endometrial cancer mortality risk increased among successive South African birth cohorts from 1924 to 1964, before declining among younger cohorts till 1998. The mortality risk of endometrial cancer mortality among each South African birth cohort is a complex interplay of risk factors and protective factors. On account of improved standard of living and urbanization, each successive South African birth cohort experienced increased prevalence of obesity, diabetes mellitus, reducing fertility rate and late age at first pregnancy [5, 16, 32, 38, 40–43]. However, the reduction in mortality risk among birth cohorts from 1964–1998 may be related to the introduction of hormonal contraceptives from 1960s. South Africa has one of the highest prevalence of contraceptive in Africa and globally as the successive South African apartheid regimes from 1960s till 1994 actively promoted and provided easy access to the injectable contraceptives in the country. Furthermore, the increased prevalence of tobacco smoking from the mid-60s in South Africa may also contributed to reduced cohort incidence and mortality RR of endometrial cancer from 1964–1998. The youngest birth cohort (1998–2003) had increased RR, possibly because of rapid epidemiological and health transition, coupled with a rapid rise in the prevalence of risk factors (obesity, Diabetes, low fertility rate) after the commencement of multi-racial democracy in 1994. The tobacco control programs and policies of the government after 1994 may also contribute to increased incidence and mortality risks of endometrial cancer among recent cohorts. There is urgent need to educate young women in South Africa about endometrial cancer risks and encourage exercise and weight reduction programs. Women with menstrual irregularity or post-menopausal bleeding should be thoroughly evaluated and government should expand oncological services in the country to cater for the huge future burden of endometrial cancer in the country.

### Ethnic disparity of endometrial cancer mortality trends

There were marked differences in the ethnic trends of endometrial cancer mortality in South Africa. Despite having a relatively high incidence rate, the mortality rates among the White ethnic group (0.66 per 100,000 women), was about half of the mortality rates among other non-white ethnic groups. Furthermore, the White ethnic group had the highest surrogate 5-year survival rate of about 91.5% (0.66 vs 7.7 per 100,000 women, MIR:0.086), followed by Indian/Asians with survival rate of 82% (1.69 vs 9.16 per 100,000 women, MIR: 0.18) and Coloureds, 77% (1.39 Vs 5.99 per 100,000 women, MIR:0.23). Blacks had the worst estimated survival rate of 64% (1.63 vs 4.5 per 100,000 women), despite having the lowest incidence of endometrial cancer in the country. Similar ethnic disparity occurred in USA as Whites had the highest incidence while Blacks had the highest mortality rate of endometrial cancer [44, 45]. The major drivers of endometrial cancer (Obesity and low parity) are higher among South African Whites and Indian/Asians as compared to the Blacks and Coloureds. Furthermore, Whites and Indian/Asians generally had higher awareness, present with early cancer stage and had better access to optimum oncological care. Majority of Whites and Indian/Asian have health insurance cover. The higher prevalence of fertility rate, hormonal contraceptives and tobacco smoking among Blacks and Couloreds can reduce endometrial cancer rates among them. Evidence of molecular and histological differences may also explain the racial disparity in endometrial cancer mortality in the country [46].

Our study demonstrated a rapid increase in burden of endometrial cancer mortality among Blacks and Coloureds at 7.5% per annum and 3.5% per annum respectively within the last 10 years of the study (2010–2018). This trend is possibly because of increased prosperity, fueling obesity and low fertility with poor access to prompt and optimum care among them, after the commencement of multi-racial democracy in 1994. The period mortality RR of endometrial cancer among Blacks increased from 2014–2018, while there was no significant period effect among non-Blacks. This result suggested that the various socio-economic and health system strengthening policies by the multi-racial government from 1994 had a significant period effect on the previously marginalized majority Blacks, but minimal effect on non-Blacks. The diagnosis and registration of endometrial cancer deaths among Blacks and Coloureds might have increased during the post-apartheid era because of the expansion of health services. The Indians/Asians (4.0% per annum) had an insignificant rise in mortality rates. Further research is required to unravel the current pattern of risks and trends in them. In contrast, South African Whites had declining but stable endometrial cancer mortality trends (-0.4% per annum) from 2008–2018 which is similar to the declining trends in majority of HICs. This pattern suggests that with appropriate interventions, the trends among non-Whites can be reversed in South Africa.

The cohort effect was significantly strong among only Blacks and Coloureds. There was slow increase in mortality risks among successive birth cohorts from 1924 to 1984, possibly because each successive birth cohort experienced declining fertility rate, some improvement in livelihood, increased prevalence of obesity, with no easy access to optimum oncological services during the apartheid era (before 1994). The increasing prevalence of the acceptance of hormonal birth controls (injectable contraceptives), improved educational attainment, and increased prevalence of female smokers, especially among the Couloreds, might partly explain the decline in endometrial cancer mortality risk among recent cohorts of Blacks and Coloureds from 1984 to 1998. These young birth cohorts might have benefitted from the expansion of reproductive health services during the post-apartheid era from 1994. The surge in mortality risk of endometrial cancer among the youngest birth cohort from 1999–2003 may be related to the recent geometric surge in the prevalence of obesity due to the post-apartheid prosperity, consumption of refined food, and sedentary lifestyle in the country. Interventions targeting modifiable factors and creating awareness among young Black women is essential. There were no statistically significant cohort effects among Whites and Indians/Asian. However, the young White birth cohorts from 1984 had exaggerated increased mortality risks of endometrial cancer, suggesting a resurgence in the risk of endometrial cancer and reduction in smoking rates among them. Indian/Asian birth cohorts had persistently decreased risk, possibly because of increased awareness. Further studies are therefore required.

Whites (70.97± 9.70) had the highest average age at death followed by Coloreds (68.18 ± 9.48 years) and Blacks (67.18 ±11.30 years) in 2018. Surprisingly, Indian/Asian (60.05 ± 10.93 years) had the youngest average age at death from endometrial cancer, despite having a very low MIR and declining cohort mortality risk. The background higher proportion of White women living beyond 60 years in the general population, was possibly on account of better socio-economic status, easy access to healthcare and increased awareness [20, 47]. All these background socio-economic milieu might impact on the higher survival rates among White women [47]. In contrast, Blacks had the lowest proportion of women aged 60 years and above in the general population [20]. This poor health indices might also negatively impact the survival rate of endometrial cancer among Blacks. The increased age at death from 60 years in 1999 to 67 years in 2018 among Blacks may suggest at the population level that there was some improvement in care received among them. Whites also had high age at death suggesting that the endometrial cancer had good prognosis among them.

### Strength and limitation

A strength of our study is the use of high-quality national mortality data that is stratified based on age groups and ethnicity. Another strength of the study is that this is the first study to utilize both APC and Joinpoint regression model to evaluate the national endometrial cancer mortality trends in sub-Saharan Africa.

A limitation of this study was the non-availability of data on the stage and histological types of endometrial cancer [19, 48]. Being a cross-sectional study that was conducted only in South Africa, the findings may not be generalizable to other countries. Furthermore, we utilized female mid-year population denominators aged 15 years and older but did not exclude nor correct for women who had hysterectomy as the data on hysterectomy rate per age group is not available for South Africa. Ethnic specific under-reporting of the endometrial cancer cases and deaths may be a limitation of the study. However, South Africa laws proscribe burial without making a report to appropriate authorities. Ecological fallacy may occur when interpreting and extrapolating the findings from a population-based studies to individual level.

## Conclusion

In conclusion, there was strong age, period and cohort effect on the overall endometrial cancer mortality trends. The endometrial cancer mortality trends increased by about 3.7% per annum from 1999 to 2018, largely driven by increasing cohort and period mortality risks possibly related to increasing prosperity, westernization, rise in obesity and declining fertility rate. The surrogate 5-year mortality rate of endometrial cancer was lowest among Whites and Indian/Asians and worse among Coloreds and Blacks despite the latter group having the lowest incidence rates. Blacks had rapid increase in mortality rates possibly because of increasing obesity and fertility rate during the post-apartheid period. The identified disparities and trends are very useful for designing targeted intervention.

### Brief policy implications and recommendations

Although South African government initiated breast and cervical cancer control program in 2017 [49], however based on the rising burden of endometrial cancer in the country, a comprehensive integrated reproductive health cancer control program should be initiated. Primary prevention aimed at modifiable risk factors such as obesity should be encouraged. Interventions that target ethnic burden of endometrial cancer mortality should be considered. Further research is required to unravel the ethnic disparity in the current pattern of risks and trends of Endometrial cancer. To further reduce the burden of endometrial cancer in South Africa, early detection and treatment should be promoted. Physicians should have high index of suspicion and endometrial cancer symptoms such as abnormal uterine bleeding or post-menopausal bleeding should be thoroughly investigated. Interventions to further educate women on endometrial cancer risk is imperative. Additionally, targeted efforts geared towards prevention and prompt treatment of endometrial cancer among the high risk groups should be pursued by stake holders.

## Supporting information

S1 File (DOCX)

S2 File (DOC)

S1 Data (XLSX)
