# Supplementary material for: Trends and ethnic disparity in endometrial cancer mortality in South Africa (1999–2018): A population-based Age-period-cohort and Join point regression analyses
Source: PLoS One. 2025 Jan 24;20(1):e0313487. doi: 10.1371/journal.pone.0313487 (PMC11759400; doi:10.1371/journal.pone.0313487)
Supplement: S1 File — (DOCX) [file pone.0313487.s001.docx]

**ENDOMETRIAL CANCER TRENDS.**

**SUPPLEMENTARY FIGURES S1-S5**


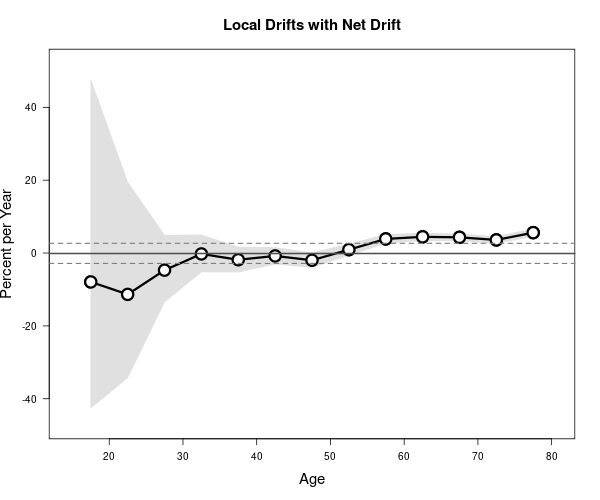

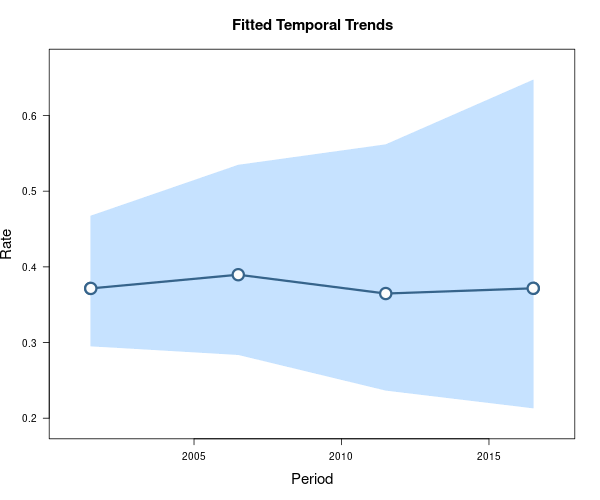

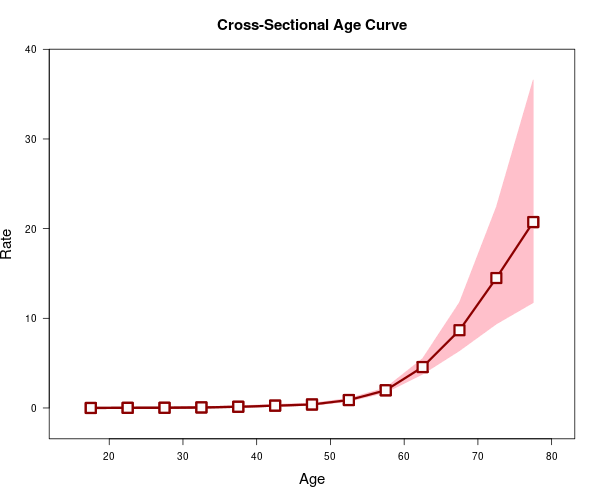

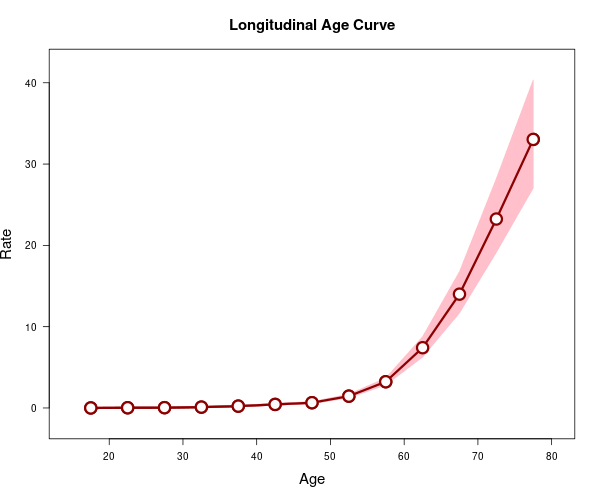

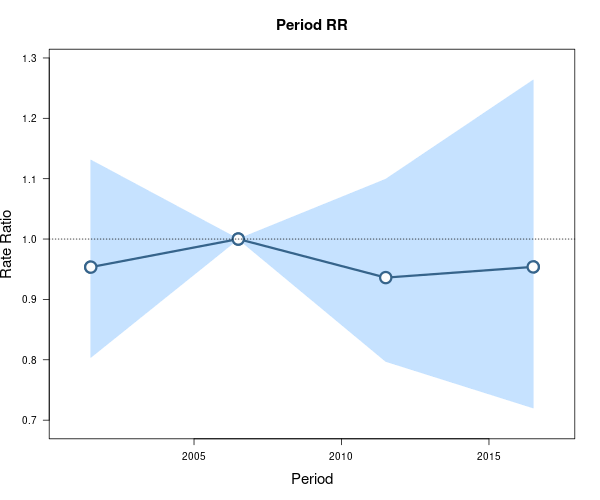

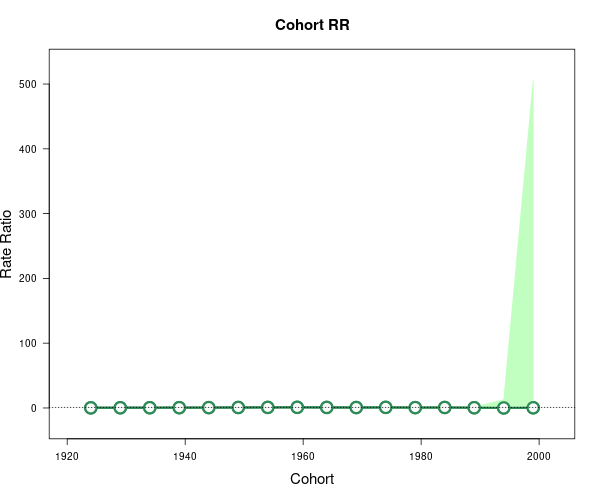


**S1 Fig**. Age, period and cohort effects of Endometrial cancer mortality in South Africa (1999-2018). **Overall** (Local drift, fitted temporal trends, longitudinal age curve, cross sectional age curve, period effect and cohort effect were depicted)


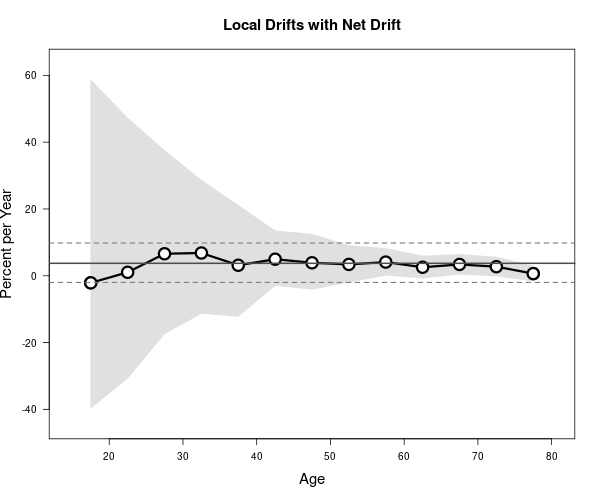

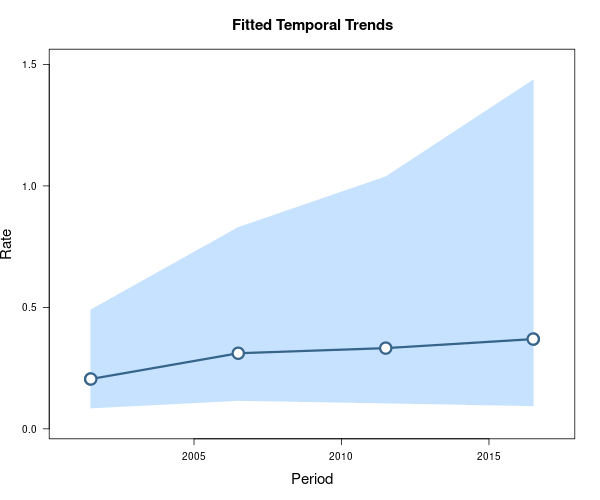

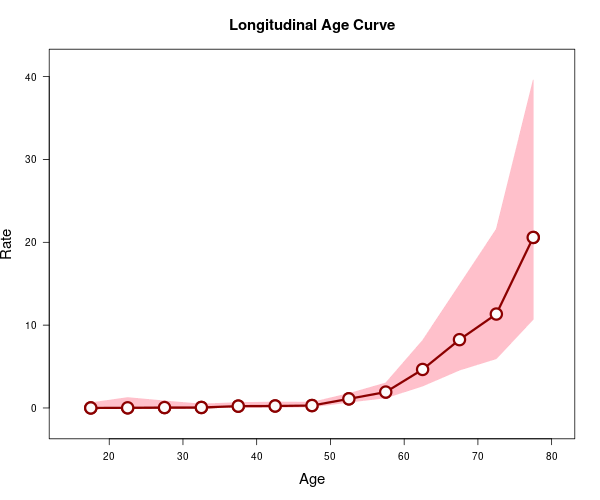

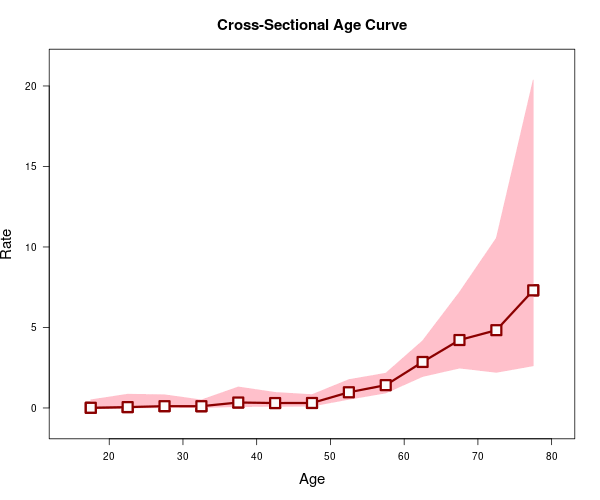

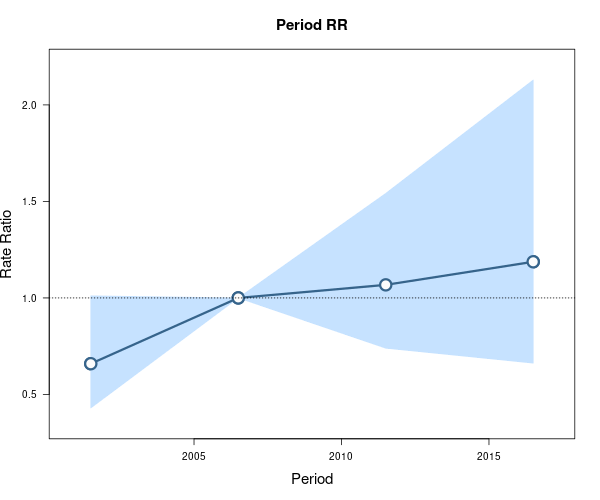

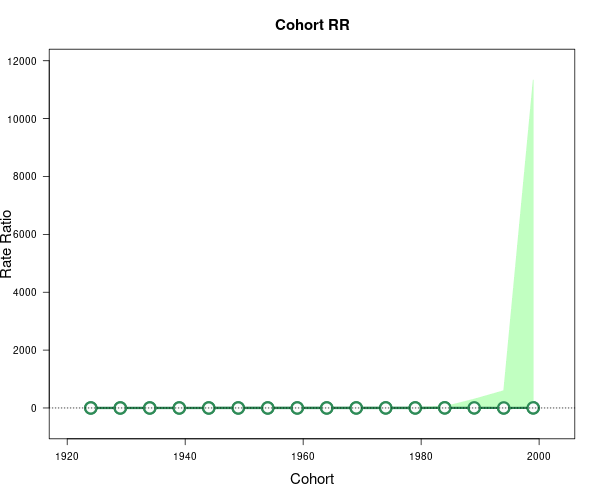


**Black**

A

**S2 Fig**. Age, period and cohort effects of Endometrial cancer mortality among **White** South Africans (1999-2018). (Local drift, fitted temporal trends, longitudinal age curve, cross sectional age curve, period effect and cohort effect were depicted


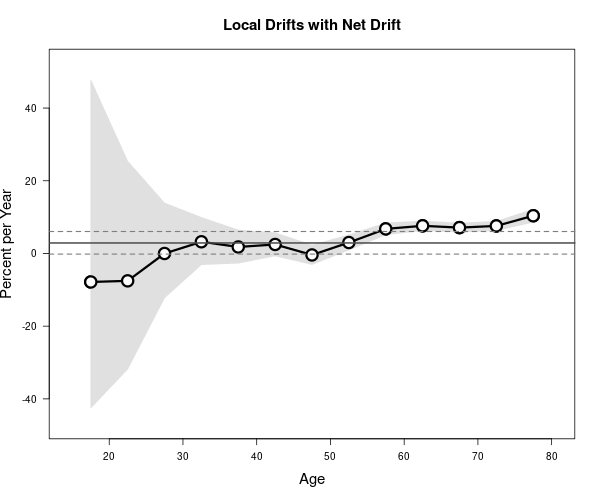


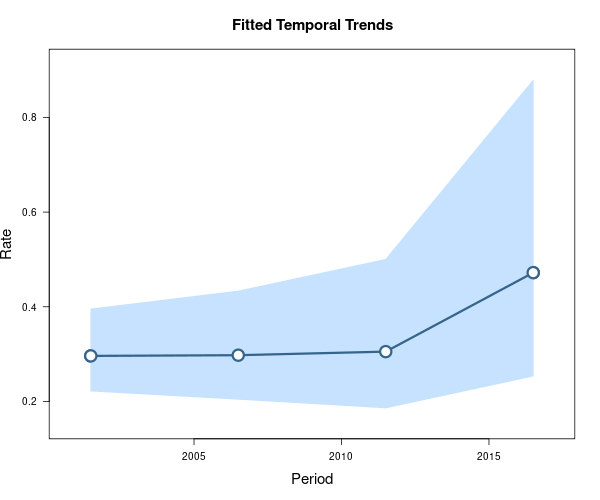

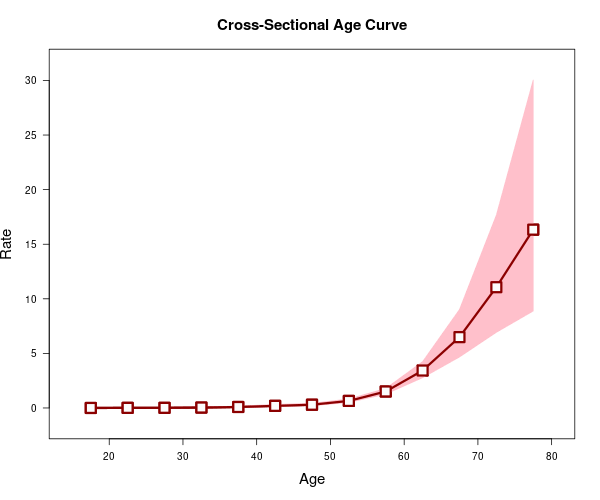

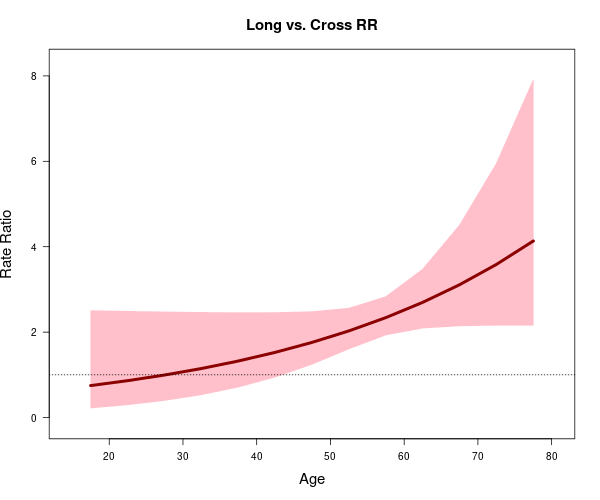

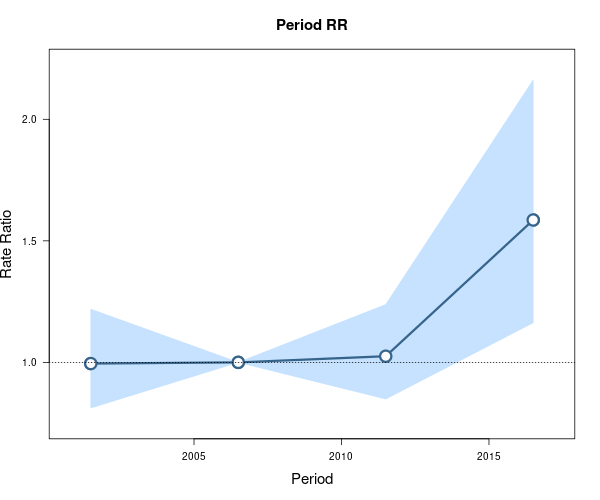

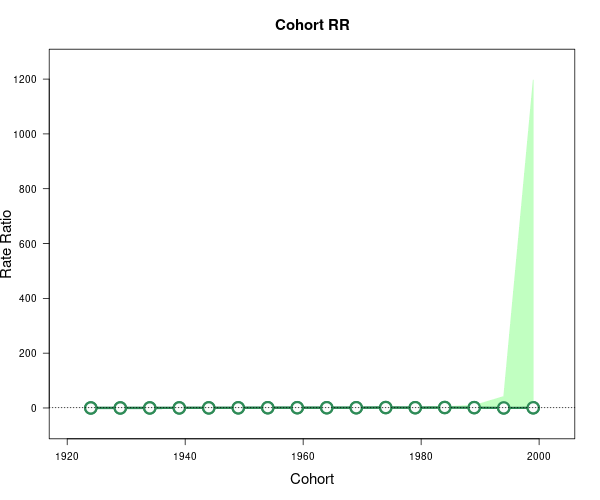


**S3 Fig**. Age, period and cohort effects of Endometrial cancer mortality among Black South Africans (1999-2018). (Local drift, fitted temporal trends, longitudinal age curve, cross sectional age curve, period effect and cohort effect were depicted)


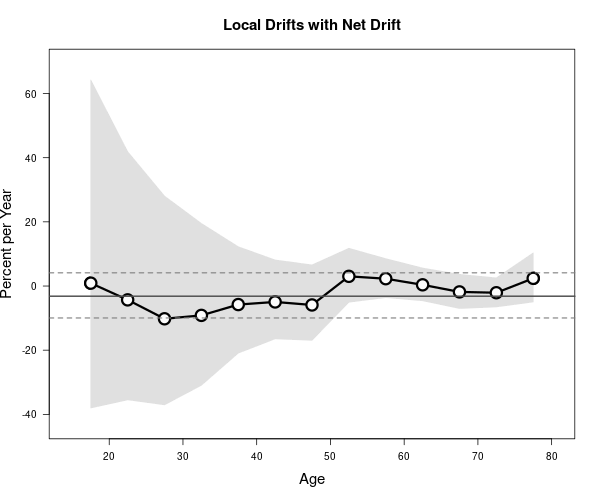

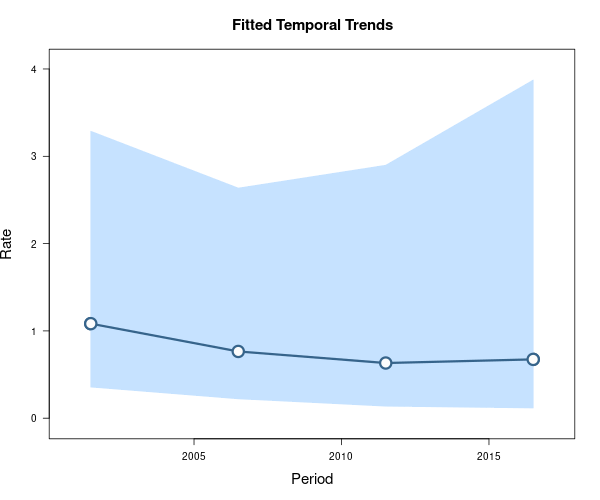

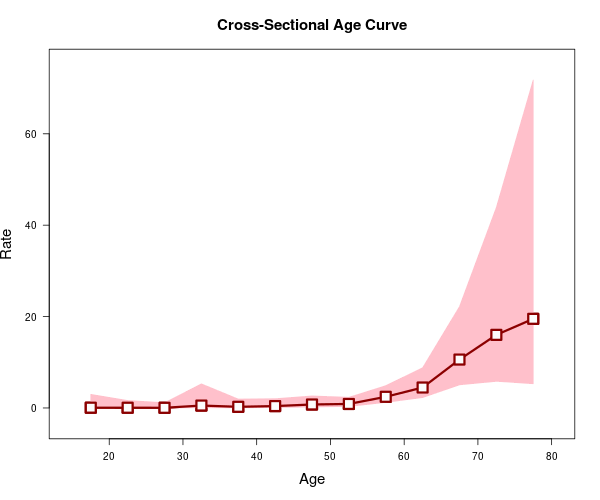

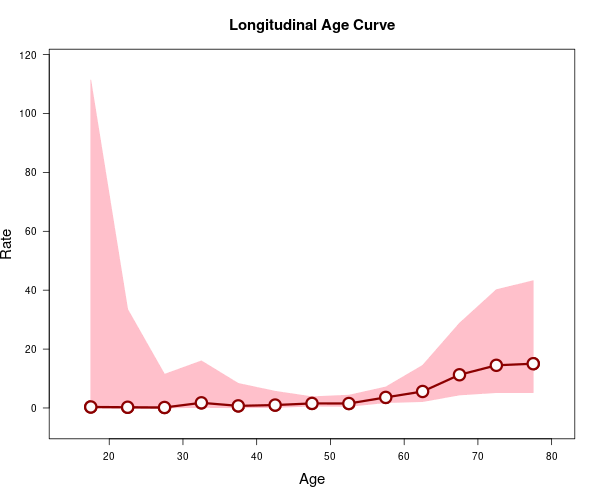

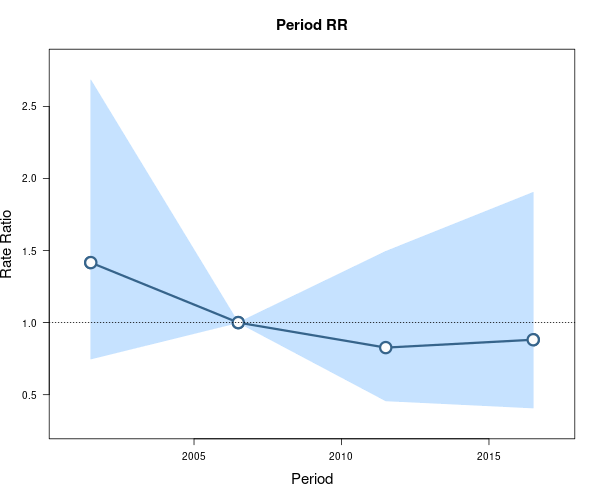

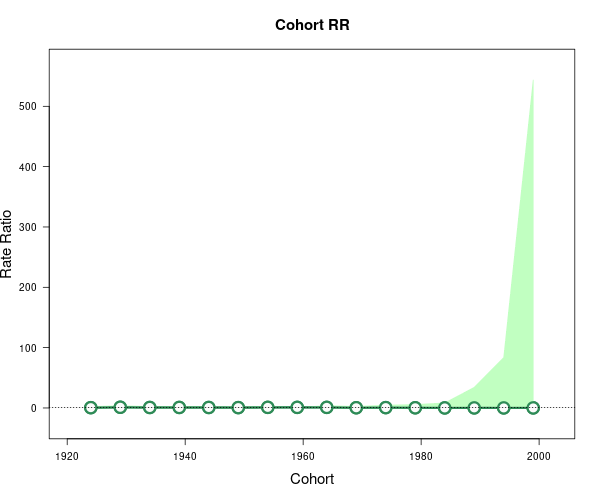


**White**

B

**S4 Fig**. Age, period and cohort effects of Endometrial cancer mortality among **Indian/Asian** South Africans (1999-2018). (Local drift, fitted temporal trends, longitudinal age curve, cross sectional age curve, period effect and cohort effect were depicted)

**Asian**

C


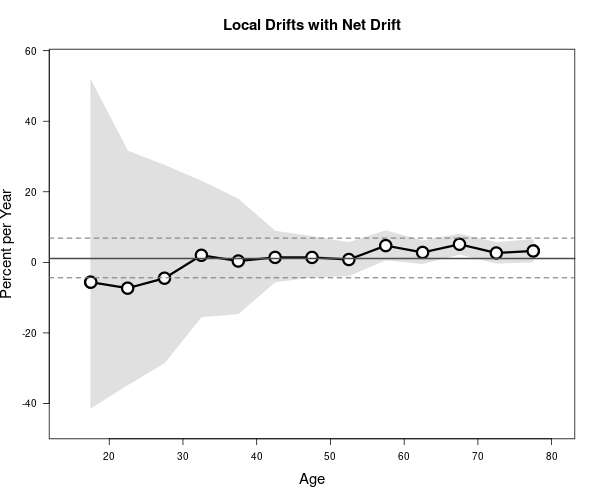

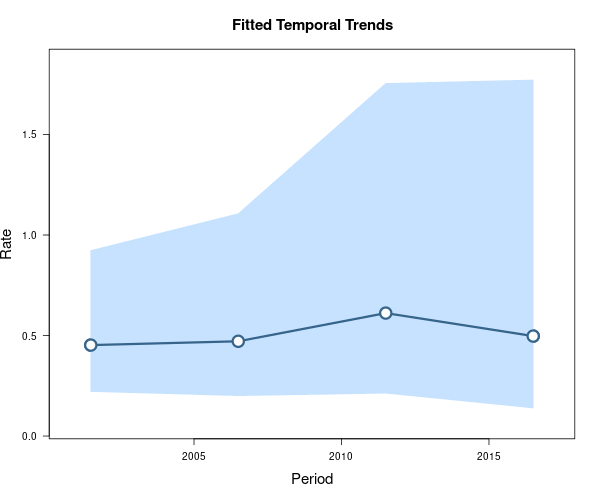

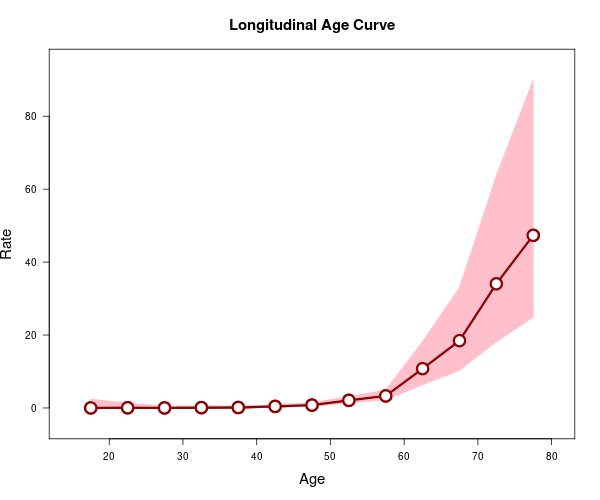

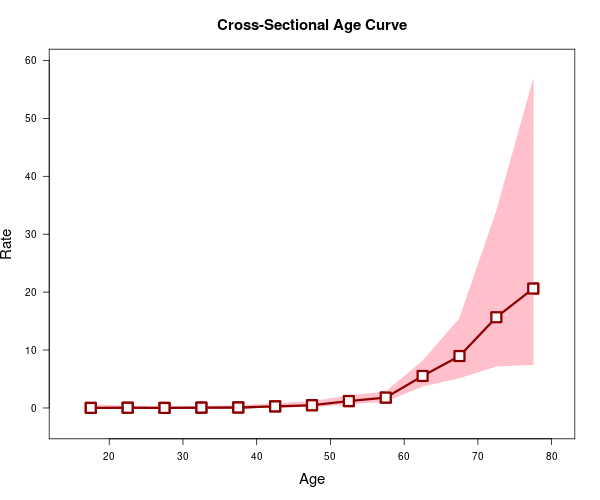

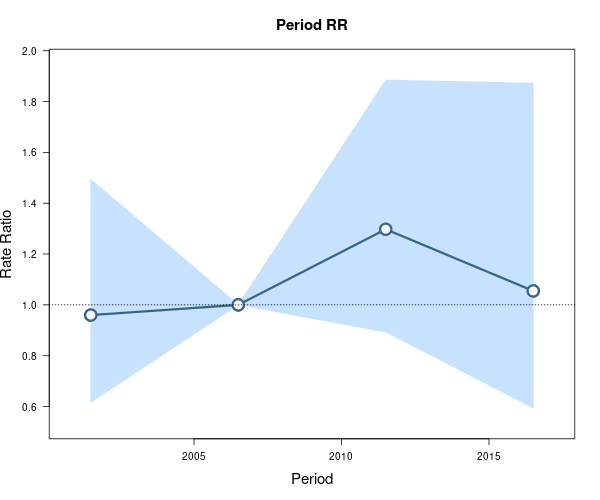

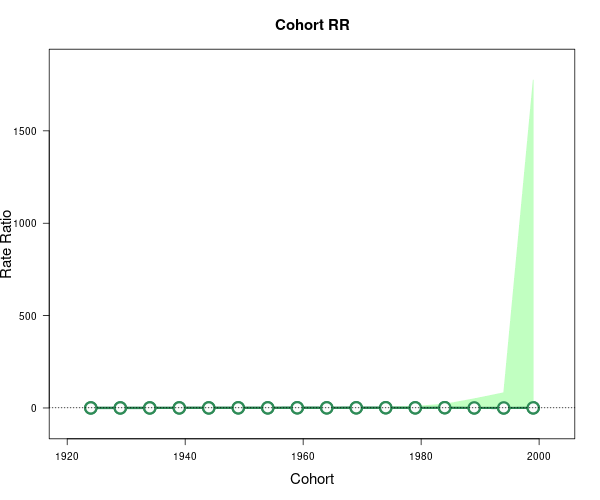


**Coloured**

D

**S5 Fig**. Age, period and cohort effects of Endometrial cancer mortality among **Coulored ethnic group** of South Africans (1999-2018). (Local drift, fitted temporal trends, longitudinal age curve, cross sectional age curve, period effect and cohort effect were depicted)
