# Supplementary material for: Trends and ethnic disparity in endometrial cancer mortality in South Africa (1999–2018): A population-based Age-period-cohort and Join point regression analyses
Source: PLoS One. 2025 Jan 24;20(1):e0313487. doi: 10.1371/journal.pone.0313487 (PMC11759400; doi:10.1371/journal.pone.0313487)
Supplement: S2 File — (DOC) [file pone.0313487.s002.doc]

S1 Table. Distribution of the endometrial cancer deaths by age group and ethnicity in South Africa (1999 – 2018)

| **Characteristics** | **Endometrium N= 4,877 (%)** |
| --- | --- |
| **Age (Mean ± SD) (Years)** | 67.21 ± 11.45 |
| 15-49 | 339 (6.95) |
| 50-69 | 2,377 (48.74) |
| ≥70 | 2,161(44.31) |
| **Ethnicity** |  |
| Blacks | 2,875(69.44) |
| White | 579(13.99) |
| Colored | 522(12.61) |
| Indian/Asians | 164 (3.96) |

S2 Table. Trends in mortality rate and mean age at death of Endometrial cancer among ethnic groups in South Africa , 1999 – 2018.

| Year | Black (n= 2,934) | | | | White (n=589) | | | | Coloured (n= 527) | | | | Asian (n= 164) | | | |
| --- | --- | --- | --- | --- | --- | --- | --- | --- | --- | --- | --- | --- | --- | --- | --- | --- |
|  | Mortality (% of gynae & breast)  ≥ 15 year | Age  mean ± SD | CMR | ASMR | Mortality (% of gynae & breast)  ≥ 15 year | Age  mean ± SD | CMR | ASMR | Mortality (% of gynae & breast)  ≥ 15 year | Age  mean ± SD | CMR | ASMR | Mortality (% of gynae & breast)  ≥ 15 year | Age  mean ± SD | CMR | ASMR |
| 1999 | 63 | 60.59±11.93 | 0.55 | 0.57 | 18 | 71.06±13.49 | 0.99 | 0.44 | 19 | 68.79±12.13 | 1.38 | 1.32 | 8 | 65.63±9.12 | 1.90 | 1.79 |
| 2000 | 67 | 65.28±12.62 | 0.56 | 0.62 | 16 | 74.13±10.58 | 0.88 | 0.36 | 10 | 64.80±11.18 | 0.70 | 0.70 | 2 | 63.00±9.90 | 0.46 | 0.42 |
| 2001 | 87 | 66.31±10.14 | 0.70 | 0.67 | 26 | 71.42±10.25 | 1.43 | 0.65 | 14 | 69.79±12.09 | 0.96 | 0.96 | 4 | 53.50±17.33 | 0.91 | 0.67 |
| 2002 | 86 | 65.09±11.71 | 0.68 | 0.64 | 17 | 74.59±10.52 | 0.92 | 0.39 | 15 | 66.47±10.34 | 1.01 | 1.04 | 9 | 63.67±13.48 | 2.01 | 1.85 |
| 2003 | 87 | 66.03±12.32 | 0.66 | 0.60 | 21 | 68.90±15.98 | 1.16 | 0.55 | 16 | 66.69±13.48 | 1.04 | 1.01 | 3 | 65.00±10.58 | 0.65 | 0.54 |
| 2004 | 105 | 65.94±13.74 | 0.86 | 0.93 | 29 | 70.10±12.33 | 1.59 | 0.73 | 13 | 65.69±11.21 | 0.89 | 0.99 | 9 | 72.00±9.73 | 2.08 | 1.73 |
| 2005 | 89 | 65.64±10.38 | 0.71 | 0.71 | 15 | 66.73±13.79 | 0.82 | 0.38 | 15 | 68.53±14.17 | 0.99 | 0.92 | 6 | 68.33±6.56 | 1.35 | 0.99 |
| 2006 | 112 | 66.19±10.75 | 0.89 | 0.88 | 23 | 67.87±10.87 | 1.25 | 0.56 | 23 | 64.48±11.13 | 1.50 | 1.37 | 11 | 56.64±12.93 | 2.43 | 1.72 |
| 2007 | 81 | 68.40±11.65 | 0.63 | 0.60 | 35 | 68.89±12.79 | 1.90 | 0.83 | 23 | 66.13±14.63 | 1.47 | 1.29 | 10 | 70.50±8.63 | 2.18 | 1.52 |
| 2008 | 120 | 64.74±11.12 | 0.89 | 0.90 | 30 | 69.90±9.74 | 1.58 | 0.71 | 19 | 65.05±14.27 | 1.15 | 1.08 | 3 | 57.33±9.61 | 0.62 | 0.44 |
| 2009 | 132 | 67.99±11.25 | 0.96 | 0.98 | 28 | 67.96±18.56 | 1.53 | 0.81 | 32 | 64.31±9.74 | 1.92 | 1.77 | 8 | 65.00±8.21 | 1.60 | 1.10 |
| 2010 | 116 | 66.52±10.81 | 0.84 | 0.87 | 28 | 69.14±9.85 | 1.45 | 0.60 | 23 | 67.57±11.36 | 1.36 | 1.19 | 6 | 68.17±7.86 | 1.18 | 0.78 |
| 2011 | 150 | 66.45±10.54 | 1.07 | 1.03 | 36 | 71.75±13.25 | 1.86 | 0.75 | 32 | 65.50±11.95 | 1.85 | 1.67 | 8 | 75.13±9.54 | 1.58 | 1.01 |
| 2012 | 146 | 67.36±10.98 | 1.03 | 0.97 | 29 | 69.66±12.16 | 1.48 | 0.60 | 28 | 66.18±10.34 | 1.60 | 1.45 | 5 | 69.00±5.83 | 0.97 | 0.70 |
| 2013 | 187 | 66.43±10.29 | 1.23 | 1.07 | 37 | 70.05±14.10 | 1.89 | 0.75 | 50 | 68.42±11.07 | 2.76 | 2.39 | 10 | 68.20±12.79 | 1.93 | 1.35 |
| 2014 | 227 | 68.33±10.43 | 1.48 | 1.65 | 43 | 69.47±12.76 | 2.22 | 0.32 | 28 | 68.04±10.39 | 1.54 | 1.45 | 12 | 68.50±7.01 | 2.30 | 1.57 |
| 2015 | 238 | 68.18±10.35 | 1.54 | 1.42 | 33 | 65.85±14.05 | 1.70 | 0.73 | 38 | 65.53±13.13 | 2.04 | 1.59 | 14 | 65.36±10.78 | 2.63 | 1.65 |
| 2016 | 213 | 68.49±10.39 | 1.34 | 1.26 | 36 | 71.22±11.02 | 1.86 | 0.67 | 48 | 68.58±11.68 | 2.60 | 2.14 | 9 | 65.89±12.30 | 1.66 | 1.01 |
| 2017 | 274 | 67.23±10.79 | 1.69 | 1.58 | 41 | 73.00±8.78 | 2.11 | 0.70 | 38 | 68.95±11.44 | 2.03 | 1.42 | 10 | 72.60±8.69 | 1.80 | 0.78 |
| 2018 | 295 | 67.18±11.30 | 1.78 | 1.64 | 38 | 70.97±9.70 | 1.93 | 0.66 | 38 | 68.18±9.48 | 1.97 | 1.31 | 17 | 60.53±10.93 | 2.92 | 1.69 |

S3 Table. Join point estimates of the national trends in age specific mortality rates for enodemetrial cancers in South Africa , 1999 – 2018

| **Endometrium** | **Range** | **Lower Endpoint** | **Upper Endpoint** | **AAPC** | **Lower CI** | **Upper CI** | **Test Statistic~** | **P-Value~** |
| --- | --- | --- | --- | --- | --- | --- | --- | --- |
| 15-19 | - | - | - |  |  |  |  |  |
| 20-24 | Full Range | 1999 | 2018 | -8.1 | -24.3 | 11.5 | -0.9 | 0.4 |
| 25-29 | 1 | 1999 | 2008 | 81.4* | 9.0 | 201.6 | 2.5 | 0.0 |
|  | 2 | 2008 | 2018 | -3.3 | -11.6 | 5.9 | -0.8 | 0.4 |
|  | Full Range | 1999 | 2018 | 5.2 | -5.9 | 17.6 | 0.9 | 0.4 |
| 30-34 | 1 | 1999 | 2001 | 63.6 | -99.9 | 278825.3 | 0.1 | 0.9 |
|  | 2 | 2001 | 2018 | -3.9* | -7.5 | -0.3 | -2.3 | 0.0 |
|  | Full Range | 1999 | 2018 | -2.9 | -6.2 | 0.5 | -1.8 | 0.1 |
| 35-39 | 1 | 1999 | 2003 | 26.7 | -12.6 | 83.5 | 1.4 | 0.2 |
|  | 2 | 2003 | 2018 | -6.6* | -11.6 | -1.3 | -2.6 | 0.0 |
|  | Full Range | 1999 | 2018 | -3.4 | -7.3 | 0.7 | -1.8 | 0.1 |
| 40-44 | 1 | 1999 | 2001 | -37.3 | -77.4 | 74.1 | -1.0 | 0.3 |
|  | 2 | 2001 | 2018 | 3.2 | -0.7 | 7.2 | 1.8 | 0.1 |
|  | Full Range | 1999 | 2018 | 0.1 | -3.2 | 3.4 | 0.0 | 1.0 |
| 45-49 | 1 | 1999 | 2006 | 2.3 | -11.3 | 18.0 | 0.3 | 0.7 |
|  | 2 | 2006 | 2018 | -4.5 | -10.9 | 2.4 | -1.4 | 0.2 |
|  | Full Range | 1999 | 2018 | -2.3 | -5.7 | 1.3 | -1.3 | 0.2 |
| 50-54 | 1 | 1999 | 2011 | 4.3* | 0.2 | 8.5 | 2.3 | 0.0 |
|  | 2 | 2011 | 2018 | -3.3 | -10.6 | 4.5 | -0.9 | 0.4 |
|  | Full Range | 1999 | 2018 | 1.7 | -0.3 | 3.7 | 1.8 | 0.1 |
| 55-59 | 1 | 1999 | 2001 | -10.3 | -57.6 | 89.9 | -0.3 | 0.8 |
|  | 2 | 2001 | 2018 | 3.6* | 1.2 | 6.2 | 3.1 | 0.0 |
|  | Full Range | 1999 | 2018 | 3.0* | 1.2 | 4.9 | 3.5 | 0.0 |
| 60-64 | Full Range | 1999 | 2018 | 4.8* | 3.4 | 6.2 | 7.4 | 0.0 |
| 65-69 | 1 | 1999 | 2008 | 5.0 | -0.7 | 11.1 | 1.9 | 0.1 |
|  | 2 | 2008 | 2012 | -3.7 | -26.9 | 26.7 | -0.3 | 0.8 |
|  | 3 | 2012 | 2018 | 9.7* | 0.9 | 19.2 | 2.4 | 0.0 |
|  | Full Range | 1999 | 2018 | 3.7* | 2.2 | 5.1 | 5.4 | 0.0 |
| 70-74 | 1 | 1999 | 2004 | 15.9* | 5.6 | 27.2 | 3.5 | 0.0 |
|  | 2 | 2004 | 2007 | -6.4 | -35.4 | 35.7 | -0.4 | 0.7 |
|  | 3 | 2007 | 2018 | 4.8* | 2.6 | 7.0 | 4.9 | 0.0 |
|  | Full Range | 1999 | 2018 | 4.0* | 2.6 | 5.4 | 6.1 | 0.0 |
| 75+ | 1 | 1999 | 2004 | 18.6* | 5.6 | 33.2 | 3.1 | 0.0 |
|  | 2 | 2004 | 2018 | 2.3* | 0.5 | 4.1 | 2.8 | 0.0 |
|  | Full Range | 1999 | 2018 | 4.3* | 2.4 | 6.2 | 4.8 | 0.0 |

S4 Table. Trends in the overall and ethnic age specific rates of Endometrial cancers and in South Africa, 2018

| **Age (Year)** | **15-19** | **20-24** | **25-29** | **30-34** | **35-39** | **40-44** | **45-49** | **50-54** | **55-59** | **60-64** | **65-69** | **70-74** | **≥75** |
| --- | --- | --- | --- | --- | --- | --- | --- | --- | --- | --- | --- | --- | --- |
| **Endometrium** | | | | | | | | | | | | | |
| Overall | 0 | 0 | 0 | 0.08 | 0.19 | 0.53 | 0.60 | 1.64 | 3.41 | 7.65 | 11.14 | 13.39 | 14.51 |
| Blacks | 0.005 | 0.02 | 1.89 | 0.40 | 1.06 | 1.65 | 2.43 | 3.08 | 3.05 | 3.61 | 3.91 | 6.19 | 6.66 |
| Whites | 0.008 | 0.03 | 0.33 | 0.60 | 1.27 | 1.38 | 1.55 | 1.84 | 1.53 | 1.55 | 1.30 | 1.62 | 1.33 |
| Indian/Asians | 0.238 | 0.81 | 0.76 | 1.44 | 3.22 | 3.56 | 5.57 | 6.60 | 6.04 | 6.66 | 6.27 | 8.91 | 9.99 |
| Coloureds | 0.049 | 0.02 | 0.20 | 0.44 | 1.06 | 1.41 | 1.66 | 1.89 | 1.83 | 2.18 | 2.27 | 3.69 | 4.25 |

**S5 Table**. **Age, Period Cohort effect estimates of the national trends in Endometrial cancer mortality in South Africa, 1999-2018**.

| **Factor** | **Overall** |  |  |  |  |  |
| --- | --- | --- | --- | --- | --- | --- |
|  | **Longitudinal RR(adjusted for period effect)** | **95%CI** | **Cross sectional RR (Adjusted for cohort effect)** | **95%CI** | **Local drift(%)** | **95%CI** |
| **Age** |  |  |  |  |  |  |
| 15-19 | 0.003 | 0.000-0.150 | 0.002 | 0.000-0.050 | -7.94 | -42.54 to 47.50 |
| 20-24 | 0.03 | 0.01-0.10 | 0.02 | 0.00-0.05 | -11.36 | -34.22 to 19.45 |
| 25-29 | 0.04 | 0.01-0.11 | 0.02 | 0.01-0.06 | -4.71 | -13.43 to 4.89 |
| 30-34 | 0.10 | 0.06-0.17 | 0.06 | 0.03-0.12 | -0.25 | -5.18 to 4.94 |
| 35-39 | 0.23 | 0.16-0.32 | 0.14 | 0.08-0.24 | -1.85 | -5.23 to 1.64 |
| 40-44 | 0.44 | 0.34-0.57 | 0.26 | 0.17-0.41 | -0.80 | -3.05 to 1.50 |
| 45-49 | 0.65 | 0.53-0.79 | 0.39 | 0.28-0.53 | -1.98 | -3.97 to 0.04 |
| 50-54 | 1.45 | 1.25-1.69 | 0.88 | 0.73-1.07 | 0.92 | -0.61 to 2.48 |
| 55-59 | 3.23 | 2.83-3.68 | 1.97 | 1.73-2.25 | 3.87 | 2.66 to 5.08 |
| 60-64 | 7.41 | 6.27-8.76 | 4.56 | 3.78-5.51 | 4.45 | 3.45 to 5.46 |
| 65-69 | 14.00 | 11.69-16.77 | 8.67 | 6.39-11.76 | 4.31 | 3.38 to 5.24 |
| 70-74 | 23.24 | 19.15-28.21 | 14.49 | 9.37-22.40 | 3.60 | 2.69 to 4.51 |
| 75+ | 33.03 | 27.05-40.34 | 20.73 | 11.75-36.57 | 5.59 | 4.50 to 6.70 |
| **Net Drift (%)** |  |  |  |  | -0.13 | -2.85 to 2.67 |
| **Period** | Rate Ratio | 95%CI |  |  |  |  |
| 1999-2003 | 0.95 | 0.80-1.13 |  |  |  |  |
| 2004-2008 | 1.00 | 1.00-1.00 |  |  |  |  |
| 2009-2013 | 0.94 | 0.80-1.10 |  |  |  |  |
| 2014- 2018 | 0.95 | 0.72-1.26 |  |  |  |  |
| **Cohort** | Rate Ratio | 95%CI |  |  |  |  |
| 1924-1928 | 0.20 | 0.16-0.26 |  |  |  |  |
| 1929-1933 | 0.33 | 0.27-0.41 |  |  |  |  |
| 1934-1938 | 0.39 | 0.32-0.48 |  |  |  |  |
| 1939-1943 | 0.47 | 0.39-0.57 |  |  |  |  |
| 1944-1948 | 0.56 | 0.47-0.67 |  |  |  |  |
| 1949-1953 | 0.75 | 0.64-0.89 |  |  |  |  |
| 1954-1959 | 0.88 | 0.76-1.04 |  |  |  |  |
| 1959-1963 | 1.00 | 1.00-1.00 |  |  |  |  |
| 1964-1968 | 0.84 | 0.68-1.04 |  |  |  |  |
| 1969-1973 | 0.67 | 0.49-0.91 |  |  |  |  |
| 1974-1978 | 0.94 | 0.66-1.34 |  |  |  |  |
| 1979-1983 | 0.55 | 0.31-0.98 |  |  |  |  |
| 1984-1988 | 0.77 | 0.34-1.74 |  |  |  |  |
| 1989-1993 | 0.38 | 0.08-1.69 |  |  |  |  |
| 1994 -1998 | 0.09 | 0.00-12.80 |  |  |  |  |
| 1999-2003 | 0.31 | 0.00-506.17 |  |  |  |  |

**S6 Table**: Age Period Cohort effect estimates of ethnic trends in Endometrial cancer mortality in South Africa, 1999-2018.

|  | **ENDOMETRIUM** | | | | | | | | | | | |
| --- | --- | --- | --- | --- | --- | --- | --- | --- | --- | --- | --- | --- |
| **Factor** | **BLACK** | | | **WHITE** | | | **ASIAN** | | | **COLOURED** | | |
|  | **Longitudinal RR(adjusted for period effect(95%CI)** | **Cross sectional RR (Adjusted for cohort effect(95%CI)** | **Local drift(%)**  **(95%CI)** | **Longitudinal RR(adjusted for period effect(95%CI)** | **Cross sectional RR (Adjusted for cohort effect(95%CI)** | **Local drift(%)**  **(95%CI)** | **Longitudinal RR(adjusted for period effect(95%CI)** | **Cross sectional RR (Adjusted for cohort effect(95%CI)** | **Local drift(%)**  **(95%CI)** | **Longitudinal RR(adjusted for period effect(95%CI)** | **Cross sectional RR (Adjusted for cohort effect(95%CI)** | **Local drift(%)**  **(95%CI)** |
| **Age** |  |  |  |  |  |  |  |  |  |  |  |  |
| 15-19 | 0.001  (0.000-0.072) | 0.002  (0.000-0.052) | -7.843  (-42.504to47.713) | 0.004  (0.000-0.630) | 0.013  (0.000-0.507) | -2.118  (-39.595to58.611) | 0.344  (0.001-111.361) | 0.064  (0.001-2.980) | 0.891  (-38.009to64.202) | 0.017  (0.000-2.400) | 0.014  (0.000-0.532) | -5.623  (-41.277to51.679) |
| 20-24 | 0.010  (0.002-0.051) | 0.011  (0.003-0.052) | -7.532  (-31.806to25.383) | 0.021  (0.000-1.256) | 0.054  (0.004-0.856) | 1.037  (-30.659to47.220) | 0.256  (0.002-33.505) | 0.056  (0.002-1.638) | -4.316  (-35.471to41.883) | 0.058  (0.003-1.339) | 0.046  (0.006-0.383) | -7.321  (-34.716to31.569) |
| 25-29 | 0.01  (0.00-0.06) | 0.01  (0.00-0.05) | -0.01  (-12.20to13.87) | 0.05  (0.00-0.85) | 0.11  (0.02-0.82) | 6.59  (-17.37to37.50) | 0.17  (0.00-11.45) | 0.04  (0.00-1.14) | -10.21  (-37.03to28.03) | 0.01  (0.00-0.53) | 0.01  (0.00-0.23) | -4.49  (-28.47to27.51) |
| 30-34 | 0.04  (0.02-0.09) | 0.04  (0.02-0.08) | 3.21  (-3.07to9.91) | 0.06  (0.01-0.48) | 0.11  (0.02-0.50) | 6.82  (-11.27to28.59) | 1.76  (0.19-15.99) | 0.53  (0.05-5.28) | -9.15  (-30.93to19.50) | 0.09  (0.01-0.69) | 0.06  (0.01-0.29) | 2.00  (-15.44to23.05) |
| 35-39 | 0.12  (0.07-0.20) | 0.09  (0.05-0.18) | 1.79  (-2.67to6.46) | 0.22  (0.08-0.65) | 0.34  (0.09-1.31) | 3.14  (-12.14to21.08) | 0.72  (0.06-8.34) | 0.26  (0.03-1.95) | -5.78  (-20.91to12.25) | 0.13  (0.03-0.52) | 0.09  (0.02-0.42) | 0.37  (-14.57to17.93) |
| 40-44 | 0.30  (0.21-0.42) | 0.19  (0.12-0.32) | 2.47  (-067to5.71) | 0.24  (0.08-0.72) | 0.31  (0.10-0.97) | 4.95  (-2.90to13.44) | 0.99  (0.17-5.68) | 0.42  (0.09-2.03) | -4.96  (-16.46to8.12) | 0.43  (0.20-0.91) | 0.28  (0.10-0.77) | 1.41  (-5.53to8.85) |
| 45-49 | 0.52  (0.40-0.68) | 0.30  (0.20-0.43) | -0.41  (-3.04to2.29) | 0.29  (0.12-0.69) | 0.31  (0.12-0.83) | 3.88  (-4.02to12.44) | 1.55  (0.63-3.81) | 0.76  (0.22-2.64) | -5.90  (-16.92to6.58) | 0.78  (0.42-1.45) | 0.47  (0.20-1.11) | 1.40  (-4.20to7.32) |
| 50-54 | 1.31  (1.07-1.61) | 0.65  (0.50-0.83) | 3.01  (0.96to5.10) | 1.10  (0.69-1.75) | 0.98  (0.54-1.76) | 3.39  (-1.95to9.03) | 1.52  (0.53-4.35) | 0.88  (0.33-2.34) | 3.02  (-5.04to11.77) | 2.09  (1.39-3.15) | 1.19  (0.67-2.12) | 0.80  (-3.79to5.61) |
| 55-59 | 3.53  (3.00-4.16) | 1.51  (1.27-1.79) | 6.76  (5.16to8.39) | 1.92  (1.22-3.02) | 1.42  (0.93-2.16) | 4.09  (0.18to8.16) | 3.59  (1.81-7.13) | 2.45  (1.22-4.89) | 2.28  (-3.60to8.53) | 3.31  (2.22-4.93) | 1.79  (1.16-2.77) | 4.75  (0.69to8.97) |
| 60-64 | 9.25  (7.46-11.48) | 3.43  (2.77-4.25) | 7.61  (6.30to8.92) | 4.65  (2.65-8.16) | 2.86  (1.96-4.17) | 2.55  (-0.69to5.89) | 5.60  (2.17-14.47) | 4.48  (2.28-8.81) | 0.38  (-4.59to5.61) | 10.78  (6.37-18.24) | 5.53  (3.78-8.08) | 2.81  (-0.38to6.10) |
| 65-69 | 20.18  (15.95-25.53) | 6.49  (4.67-9.02) | 7.11  (5.88to8.37) | 8.25  (4.57-14.86) | 4.22  (2.48-7.19) | 3.38  (0.45to6.40) | 11.28  (4.42-28.78) | 10.60  (5.06-22.19) | -1.83  (-7.00to3.62) | 18.45  (10.32-33.01) | 8.96  (5.21-15.42) | 5.12  (2.30to8.01) |
| 70-74 | 39.66  (30.86-50.97) | 11.06  (6.93-17.67) | 7.58  (6.32to8.84) | 11.34  (5.95-21.59) | 4.83  (2.21-10.55) | 2.71  (-0.07to5.57) | 14.49  (5.22-40.17) | 16.00  (5.83-43.89) | -2.09  (-6.51to2.53) | 34.05  (18.18-63.77) | 15.66  (7.24-33.90) | 2.65  (-0.25to5.63) |
| 75+ | 67.51  (52.04-87.56) | 16.33  (8.88-30.04) | 10.37  (8.62to12.16) | 20.59  (10.70-39.59) | 7.31  (2.62-20.37) | 0.64  (-1.58to2.91) | 15.03  (5.23-43.23) | 19.51  (5.30-71.77) | 2.39  (-4.96to10.31) | 47.34  (24.92-89.92) | 20.62  (7.51-56.62) | 3.23  (0.08to6.47) |
| **Net Drift (%)** |  |  | 2.89  (-0.17to6.04) |  |  | 3.73  (-2.01to9.80) |  |  | -3.18  (-9.96to4.12) |  |  | 1.09  (-4.37to6.87) |
| **Period** | Rate Ratio  (95%CI) |  |  | Rate Ratio  (95%CI) |  |  | Rate Ratio  (95%CI) |  |  | Rate Ratio  (95%CI) |  |  |
| 1999-2003 | 1.00  (0.81-1.22) |  |  | 0.66  (0.43-1.01) |  |  | 1.42  (0.75-2.68) |  |  | 0.96  (0.62-1.49) |  |  |
| 2004-2008 | 1.00  (1.00-1.00) |  |  | 1.00  (1.00-1.00) |  |  | 1.00  (1.00-1.00) |  |  | 1.00  (1.00-1.00) |  |  |
| 2009-2013 | 1.03  (0.85-1.24) |  |  | 1.07  (0.74-1.54) |  |  | 0.83  (0.46-1.49) |  |  | 1.30  (0.89-1.88) |  |  |
| 2014- 2018 | 1.59  (1.16-2.16) |  |  | 1.19  (0.66-2.13) |  |  | 0.88  (0.41-1.90) |  |  | 1.05  (0.59-1.87) |  |  |
| **Cohort** | Rate Ratio  (95%CI) |  |  | Rate Ratio  (95%CI) |  |  | Rate Ratio  (95%CI) |  |  | Rate Ratio  (95%CI) |  |  |
| 1924-1928 | 0.06  (0.05-0.09) |  |  | 0.43  (0.21-0.85) |  |  | 0.51  (0.11-2.40) |  |  | 0.28  (0.13-0.59) |  |  |
| 1929-1933 | 0.13  (0.10-0.17) |  |  | 0.31  (0.15-0.63) |  |  | 1.19  (0.39-3.65) |  |  | 0.32  (0.15-0.65) |  |  |
| 1934-1938 | 0.20  (0.15-0.26) |  |  | 0.42  (0.21-0.83) |  |  | 0.81  (0.27-2.46) |  |  | 0.32  (0.17-0.62) |  |  |
| 1939-1943 | 0.29  (0.23-0.37) |  |  | 0.43  (0.23-0.80) |  |  | 0.87  (0.32-2.35) |  |  | 0.47  (0.26-0.87) |  |  |
| 1944-1948 | 0.39  (0.31-0.49) |  |  | 0.48  (0.26-0.86) |  |  | 0.82  (0.32-2.08) |  |  | 0.43  (0.24-0.79) |  |  |
| 1949-1953 | 0.56  (0.45-0.70) |  |  | 0.71  (0.41-1.22) |  |  | 0.61  (0.25-1.49) |  |  | 0.76  (0.45-1.28) |  |  |
| 1954-1959 | 0.87  (0.71-1.06) |  |  | 0.57  (0.33-0.99) |  |  | 1.02  (0.41-2.51) |  |  | 0.62  (0.39-1.00) |  |  |
| 1959-1963 | 1.00  (1.00-1.00) |  |  | 1.00  (1.00-1.00) |  |  | 1.00  (1.00-1.00) |  |  | 1.00  (1.00-1.00) |  |  |
| 1964-1968 | 0.87  (0.66-1.16) |  |  | 1.02  (0.49-2.14) |  |  | 1.00  (0.26-3.85) |  |  | 0.74  (0.40-1.37) |  |  |
| 1969-1973 | 0.85  (0.56-1.27) |  |  | 1.07  (0.33-3.48) |  |  | 0.37  (0.05-2.50) |  |  | 0.87  (0.37-2.02) |  |  |
| 1974-1978 | 1.52  (0.95-2.43) |  |  | 2.20  (0.65-7.43) |  |  | 0.60  (0.08-4.68) |  |  | 1.20  (0.39-3.68) |  |  |
| 1979-1983 | 0.97  (0.46-2.02) |  |  | 1.34  (0.10-18.13) |  |  | 0.32  (0.02-5.68) |  |  | 0.71  (0.05-9.23) |  |  |
| 1984-1988 | 1.67  (0.59-4.70) |  |  | 3.79  (0.27-52.88) |  |  | 0.09  (0.00-8.40) |  |  | 1.44  (0.10-21.02) |  |  |
| 1989-1993 | 1.27  (0.17-9.70) |  |  | 4.52  (0.07-307.72) |  |  | 0.15  (0.00-34.26) |  |  | 0.44  (0.00-49.90) |  |  |
| 1994 -1998 | 0.29  (0.00-41.99) |  |  | 1.50  (0.00-599.43) |  |  | 0.13  (0.00-83.73) |  |  | 0.30  (0.00-82.67) |  |  |
| 1999-2003 | 0.70  (0.00-1196.59) |  |  | 3.82  (0.00-11333.16) |  |  | 0.11  (0.00-543.56) |  |  | 0.63  (0.00-1775.12) |  |  |

S7 Table. Wald Chi-square test for estimable functions of age period cohort model in the overall and ethnic trends of endometrial cancer mortality in South Africa (1999 – 2018)

| **Gynaecological cancer type** | **NetDrift = 0** | | **All Period RR = 1** | | **All Cohort RR = 1** | | **All Local Drifts = Net Drift** | |
| --- | --- | --- | --- | --- | --- | --- | --- | --- |
|  | **Chi-square** | **P-value** | **Chi-square** | **P-value** | **Chi-square** | **P-value** | **Chi-square** | **P-value** |
| **Endometrium** |  |  |  |  |  |  |  |  |
| Overall | 0.0085 | 0.9267 | 2.04 | 0.5648 | 248.58 | 2.43E-44* | 60.07 | 5.10E-08* |
| **Black** | 3.42 | 0.06 | 41.70 | 4.64E^-09*^ | 403.25 | 1.44E^-76*^ | 52.12 | 1.29E^-06*^ |
| **White** | 1.59 | 0.21 | 3.79 | 0.29 | 17.79 | 0.27 | 6.13 | 0.94 |
| **Asian** | 0.76 | 0.38 | 1.78 | 0.62 | 6.56 | 0.97 | 5.17 | 0.97 |
| **Coloured** | 0.15 | 0.70 | 5.41 | 0.14 | 27.17 | 0.03* | 12.47 | 0.49 |
| *Statistically significant at P-value< 0.05 | | | | | | | | |
